# TSPO Regulates TLR4-Mediated Inflammation Through Calcium Homeostasis and Immunometabolic Adaptation

**DOI:** 10.3390/ijms27167336

**Published:** 2026-08-17

**Authors:** Xiaoqin Wu, Yaru Zhu, Bo Liu, Xiaoni Liu, Xiangjun Chen

**Affiliations:** 1 Department of Neurology, Huashan Hospital, Fudan University and Institute of Neurology, Fudan University, National Center for Neurological Disorders, Shanghai 200040, China; 2 State Key Laboratory of Immune Response and Immunotherapy, Shanghai Institute of Materia Medica, Chinese Academy of Sciences, Shanghai 201203, Chinabliu@ips.ac.cn (B.L.); 3 University of Chinese Academy of Sciences, Beijing 101408, China

**Keywords:** TSPO, TLR4, LPS, inflammation, metabolic reprogramming

## Abstract

Bacterial infection triggered excessive inflammatory responses, yet the mechanisms linking inflammatory activation to immunometabolic adaptation remained incompletely understood. The mitochondrial translocator protein (TSPO) has been implicated in inflammatory activation and cellular metabolism. This study aimed to investigate the role of TSPO in inflammation mediated by Toll-like receptor 4 (TLR4). Herein, we integrated transcriptomic data from the human peripheral blood dataset GSE72829, and single-cell transcriptomic profiles from the CELLxGENE platform with cellular mechanistic experiments in BV2 microglia and RAW264.7 macrophages. Transcriptomic analyses revealed that *TSPO* expression was markedly upregulated in patients with bacterial infection (*n* = 52) and exhibited diagnostic potential to distinguish bacterial infection from healthy controls (HCs, *n* = 16) and viral infection (*n* = 92). *TSPO*-correlated genes were enriched in Toll-like receptor (TLR) signaling, inflammatory response, and immunometabolic pathways. Mechanistically, TSPO interacted with TLR4 and selectively modulated TLR4-driven inflammatory activation. TSPO deficiency augmented lipopolysaccharide (LPS) induced tumor necrosis factor‑α (TNF-α) and interleukin‑6 (IL-6) secretion, accompanied by disrupted Ca^2+^ homeostasis, impaired cholesterol balance, and compensatory metabolic remodeling characterized by elevated L-lactate and sustained Adenosine triphosphate (ATP) levels. Collectively, these findings identified TSPO as an immunometabolic regulator bridging TLR4 signaling and metabolic adaptation during inflammatory activation. Besides, TSPO represented a promising biomarker and therapeutic target to limit exaggerated inflammatory responses.

## 1. Introduction

Bacterial infection remains a major global health challenge and is associated with substantial morbidity and mortality [1]. Recognition of bacterial pathogens is primarily mediated by innate immune receptors, among which Toll-like receptor 4 (TLR4) serves as a major sensor for lipopolysaccharide (LPS) derived from Gram-negative bacteria. Upon activation, TLR4 activates nuclear factor-κB (NF-κB) and mitogen-activated protein kinase (MAPK) signaling pathways, leading to the production of inflammatory mediators, including interleukin-6 (IL-6) and tumor necrosis factor-α (TNF-α), which are essential for host defense but may become detrimental when excessively sustained [2,3]. Therefore, maintaining an appropriate balance between inflammatory activation and regulatory adaptation is essential during bacterial infection. Although extensive studies have characterized canonical TLR4 signaling pathways, the molecular mechanisms that integrate TLR4 activation with cellular stress responses and metabolic adaptation remain incompletely understood.

Immune activation is accompanied by extensive metabolic remodeling to meet the increased energetic and biosynthetic demands of inflammatory responses [4,5]. During bacterial infection, immune cells undergo coordinated changes in mitochondrial function, lipid metabolism, reactive oxygen species (ROS) production, and energy utilization [6]. In parallel, compensatory protective programs, including induction of anti-inflammatory mediators such as interleukin-10 (IL-10) and arginase 1 (Arg-1), as well as antioxidant pathways mediated by heme oxygenase-1 (HMOX1), are activated to restrict excessive inflammation and preserve cellular homeostasis [7,8]. Mitochondria represent a critical hub integrating inflammatory signaling and metabolic adaptation; however, the mitochondrial-associated regulators that link inflammatory responses with immunometabolic remodeling remain largely unknown.

The translocator protein 18 kDa (TSPO), an evolutionarily conserved mitochondrial outer membrane protein, is highly expressed in activated microglia and macrophages and has been widely used as a marker of inflammation [9]. Beyond its diagnostic application, TSPO participates in multiple mitochondrial processes, including cholesterol transport, calcium (Ca^2+^) homeostasis, and cellular metabolism [10]. Mitochondrial Ca^2+^ signaling is increasingly recognized as an important regulator of immune activation, as alterations in Ca^2+^ homeostasis can influence inflammatory signaling cascades and metabolic reprogramming [11,12]. However, how TSPO integrates TLR4-mediated inflammatory signaling with mitochondrial homeostasis and metabolic adaptation has not been elucidated.

In this study, we investigated the role of TSPO in TLR4 signaling using integrated transcriptomic analyses from public clinical datasets, and CELLxGENE platforms, together with mechanistic studies in BV2 microglia and RAW 264.7 macrophage models. First, we identified TSPO as a bacterial infection-associated signature and evaluated its diagnostic performance. Furthermore, we demonstrated that TSPO was closely associated with TLR4-related inflammatory networks and immunometabolic pathways. Functional studies revealed that TSPO selectively regulated TLR4-mediated inflammation by modulating mitochondrial Ca^2+^ handling, cholesterol homeostasis, and cellular metabolic adaptation. Collectively, our findings identified TSPO as an immunometabolic regulator linking innate immune signaling with mitochondrial adaptation during inflammatory activation.

## 2. Results

### 2.1. TSPO and Components of the TLR4 Signaling Pathway Were Upregulated in Patients with Definite Bacterial Infection

The GSE72829 dataset was derived from the Immunopathology of Respiratory, Inflammatory and Infectious Disease Study (IRIS) [13]. This prospective cohort recruited febrile children (<17 years; axillary temperature ≥ 38 °C) requiring blood testing from hospitals in the United Kingdom, Spain, the Netherlands, and the United States between 2009 and 2013. Definite bacterial infection was defined by the identification of culture-confirmed bacterial pathogens from normally sterile sites, whereas definite viral infection was determined by positive viral detection through culture, molecular assays, or immunofluorescence in the absence of bacterial co-infection [13].

**Figure 1:**
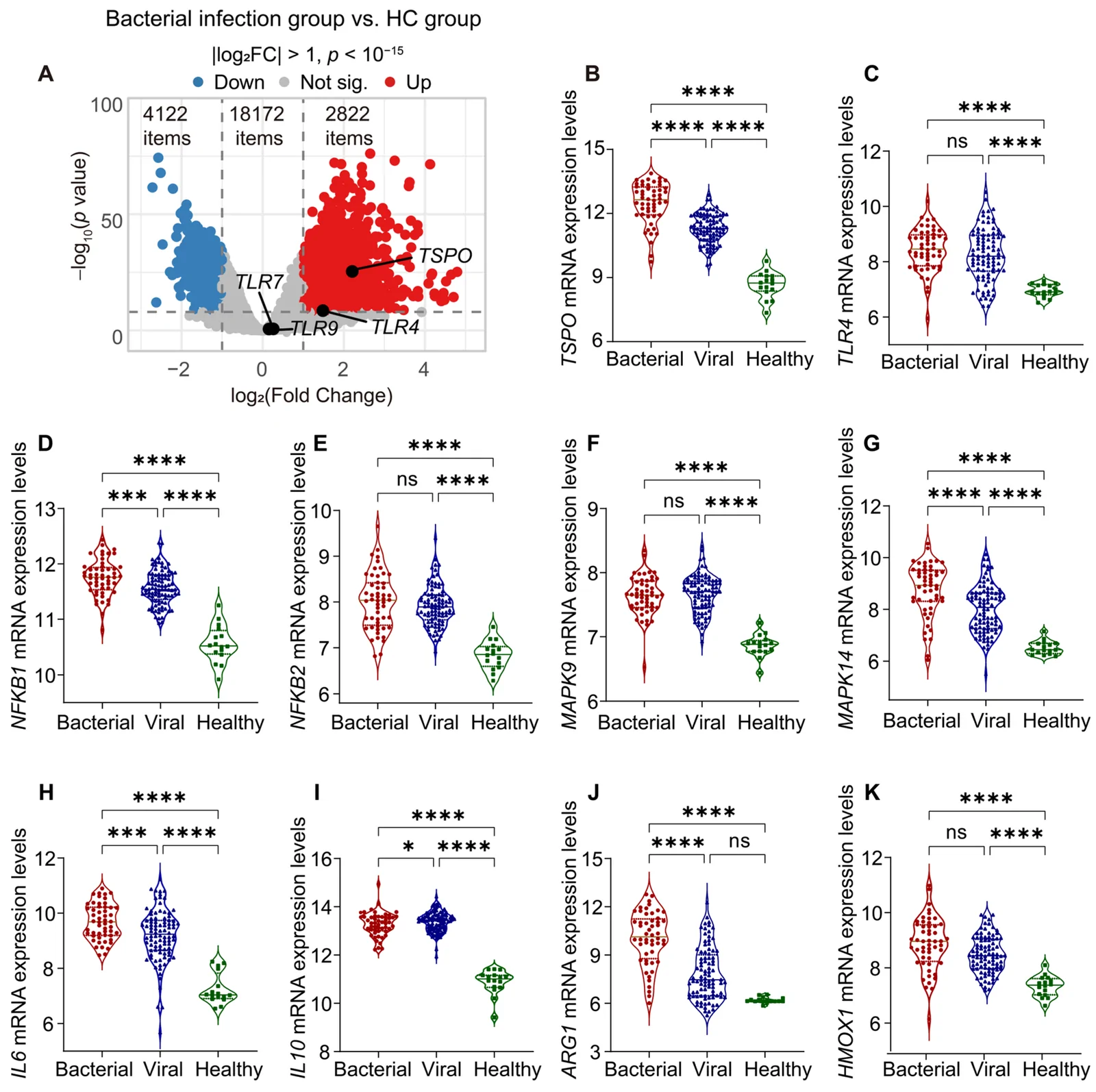
Expression profiles of TSPO and TLR4 pathway-related genes in bacterial infection. (**A**) Volcano plot showing differentially expressed genes (DEGs) between patients with bacterial infection and healthy controls (HCs). Red dots indicated significantly upregulated genes in patients with bacterial infection, with the highlights of *TSPO* and *TLR4*. (**B**–**K**) Violin plots showing the mRNA expression levels of (**B**) *TSPO*, (**C**) *TLR4*, (**D**) *NFKB1* (p50), (**E**) *NFKB2* (p52), (**F**) *MAPK9* (JNK2), (**G**) *MAPK14* (p38α), (**H**) *IL6*, (**I**) *IL10*, (**J**) *ARG1*, and (**K**) *HMOX1* among the three groups. Transcriptomic data were obtained from the GSE72829 dataset. * *p* < 0.05; *** *p* < 0.001; **** *p* < 0.0001; ns, not significant.

Analysis of the GSE72829 dataset identified 6944 differentially expressed genes (DEGs) between patients with definite bacterial infection (*n* = 52) and healthy controls (HCs; *n* = 16), comprising 2822 upregulated and 4122 downregulated genes (Figure 1A). *TSPO* was among the upregulated genes (log_2_FC = 2.208), together with *TLR4* (log_2_FC = 1.484). Comparison between bacterial and viral infection (*n* = 92) revealed a substantially smaller transcriptional difference, with 557 upregulated and 386 downregulated DEGs under the threshold of |log_2_FC| > 0.5 and *p* < 1 × 10^−5^ (Figure S1A). Notably, *TSPO* remained significantly upregulated in bacterial infection relative to viral infection (log_2_FC = 0.886). Collectively, these data identified *TSPO* as a robust transcriptional signature of bacterial infection, distinguishing it from both healthy and viral states.

As shown in Figure 1B–K, violin plot analysis further confirmed significantly elevated mRNA levels of key components in the TLR4 signaling pathway, including *TSPO*, *TLR4*, *NFKB1* (p50), *NFKB2* (p52), *MAPK9* (JNK2), and *MAPK14* (p38α), as well as the pro-inflammatory gene *IL6* in patients with bacterial infection. In addition, anti-inflammatory mediators (*IL10*, *ARG1*), and antioxidant enzyme *HMOX1* were also markedly upregulated in bacterial infection group. In contrast, no significant differences were observed in the mRNA expression levels of *TLR7*, *TLR8*, or *TLR9* between bacterial infection patients and HCs (Figure S1B–D, all *p* > 0.05).

### 2.2. Correlation Analysis of the Associations Between TSPO and Genome-Wide Expression Profiles

**Figure 2:**
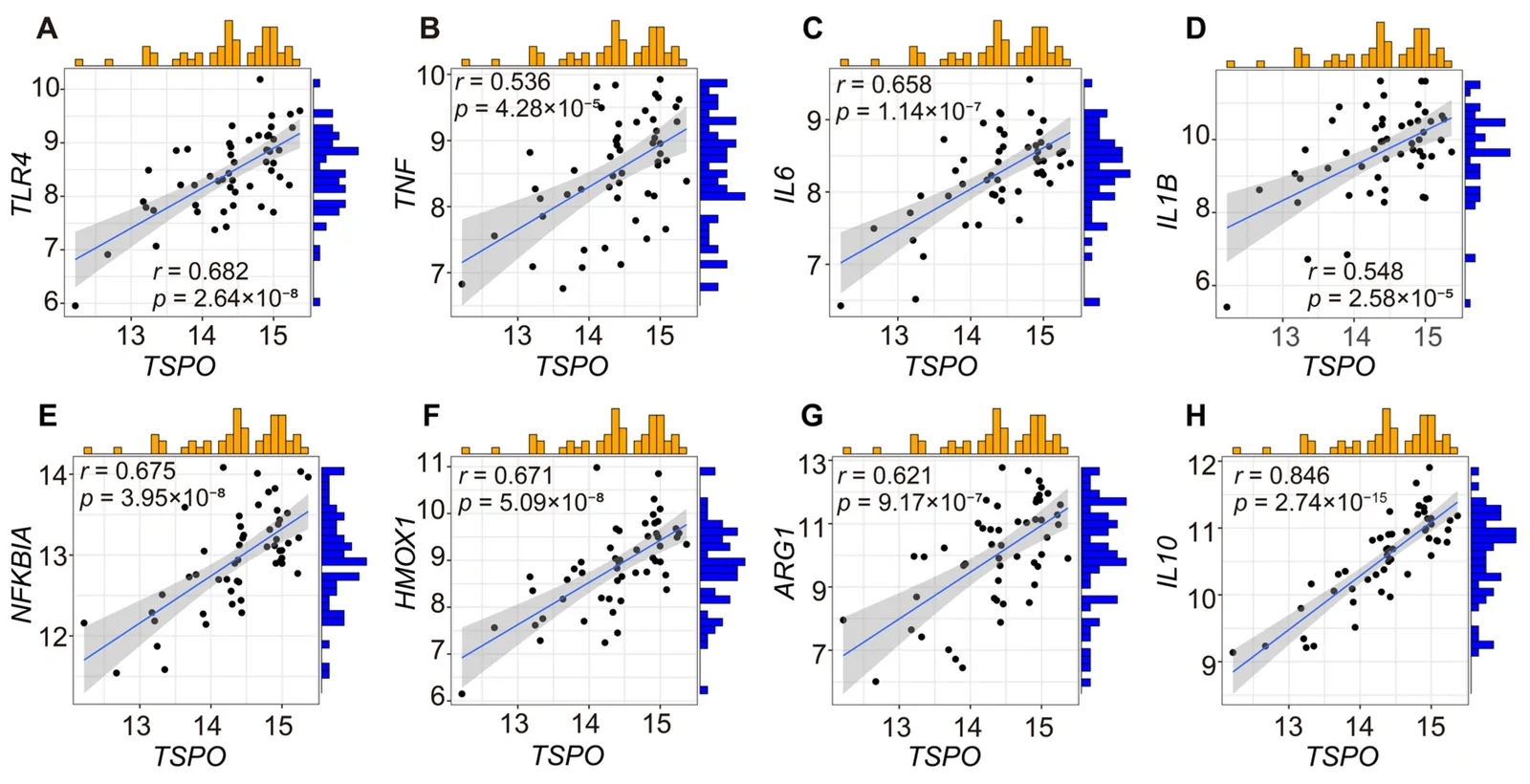
*TSPO* correlation with components of TLR4 pathways in bacterial infection (*n* = 52). (**A**–**H**) Spearman correlation analyses between *TSPO* mRNA expression and key genes of the TLR4 signaling pathways across patients with definite bacterial infection (*n* = 52). Marginal histograms (orange and blue columns) illustrating the distribution of expression levels. As shown, *TSPO* expression exhibited strongly positive correlations with (**A**) *TLR4*, (**B**) *TNF*, (**C**) *IL6*, (**D**) *IL1B*, (**E**) *NFKBIA*, (**F**) *HMOX1*, (**G**) *ARG1*, and (**H**) *IL10*. Data were obtained from the GSE72829 dataset.

To further investigate the association between *TSPO* and inflammatory signaling, Spearman correlation analyses were respectively performed in the HCs (Table S1) and bacterial infection group (Table S2 and Figure 2). In HC cohort, only *NFKBIA* (IκBα) showed a positive correlation with *TSPO* (*r* = 0.523, *p* = 0.0375), while other tested genes (*TLR4*, *TNF*, *IL6*, *CAMKK2*, *HMOX1*, *ARG1* and *IL10*) exhibited no statistically significant association with *TSPO*. In sharp contrast, robust and widespread correlations between *TSPO* and immune-related transcripts were detected in bacterial infection subjects. *TSPO* expression showed a strong positive correlation with *TLR4* (*r* = 0.682, *p* < 0.0001), whereas negative correlation was observed with *TLR7* (*r* = −0.448), and no significant correlation was detected with *TLR9* (*p* > 0.05).

Consistently, *TSPO* expression was positively correlated with downstream mediators of the *TLR4* pathway (Table S2 and Figure 2), including *NFKB1* (p50; *r* = 0.459), pro-inflammatory cytokines *TNF* (*r* = 0.536), *IL6* (*r* = 0.658), and *IL1B* (*r* = 0.548, all *p* < 0.001). Notably, strong positive correlations were also observed with anti-inflammatory mediators (Figure 2E–H), including *NFKBIA* (IκBα, *r* = 0.675), *ARG1* (*r* = 0.621) and *IL10* (*r* = 0.846), and antioxidant enzyme *HMOX1* (*r* = 0.671, all *p* < 0.0001), suggesting a protective role of *TSPO* in balancing inflammatory responses. Collectively, this striking group-specific co-expression signature highlighted that *TSPO* was tightly coupled to inflammatory network activation exclusively during bacterial infection, supporting its potential as a transcriptional biomarker for bacterial inflammatory responses.

### 2.3. Association of TSPO Expression with Calcium and ATPase-Related Regulatory Networks

Beyond inflammatory signaling cascades, Spearman correlation analysis revealed robust co-expression links between *TSPO* and genes governing adenosine triphosphatase (ATPase) activity and calcium homeostasis exclusively in patients with bacterial infection (Table S2); no such significant correlations were observed in the HC group (Table S1). Notably, *TSPO* exhibited prominent positive correlations with multiple subunits of the vacuolar ATPase (V-ATPase) complex, including *ATP6AP1* (*r* = 0.790), *ATP6V0A1* (*r* = 0.719), *ATP6V0D1* (*r* = 0.802), and *ATP6V1A* (*r* = 0.535, all *p* < 0.0001). This tight co-expression pattern suggested that *TSPO* may participate in regulating lysosomal acidification and vesicular trafficking during bacterial inflammatory responses.

Furthermore, *TSPO* was strongly correlated with core calcium signaling mediators. Specifically, *TSPO* displayed strong positive correlations with *CAMKK2* (*r* = 0.663) but a negative correlation with *CAMK2D* (*r* = −0.386, both *p* < 0.0001), highlighting a potential role of *TSPO* in modulating Ca^2+^-dependent signaling cascades. Taken together, these bacterial infection-specific transcriptional associations suggested that *TSPO* may act as a central molecular hub coordinating mitochondrial homeostasis, lysosomal function and calcium signal transduction, thereby linking inflammatory responses to metabolic regulation.

### 2.4. Concurrent Activation of Pro-Inflammatory, Metabolic, and Stress-Response Pathways in Bacterial Infection

To characterize transcriptional pathway alterations associated with bacterial infection, single-sample gene set enrichment analysis (ssGSEA) was performed using Molecular Signatures Database (MSigDB) Hallmark gene sets in both the bacterial infection and HC groups. Compared with HCs, patients with bacterial infection exhibited broad enrichment of inflammatory transcriptional programs (Figure S2A). Specifically, ssGSEA scores of inflammatory response, IL-6/JAK/STAT3 signaling, and TNF-α signaling via NF-κB were all significantly increased (all *p* < 0.001), indicating a prominent inflammatory signature associated with bacterial infection.

Beyond inflammatory pathways, several metabolic and stress-response pathways were also significantly enriched in patients with bacterial infection (Figure S2B), including the reactive oxygen species (ROS) pathway (*p* < 0.0001), xenobiotic metabolism (*p* < 0.001), and the p53 pathway (*p* < 0.0001). These findings indicated that bacterial infection was characterized by coordinated transcriptional remodeling involving inflammatory responses, oxidative stress, and cellular stress-adaptation programs.

### 2.5. Associated of TSPO Expression with Coordinated Inflammatory and Metabolic Pathway Remodeling During Bacterial Infection

**Figure 3:**
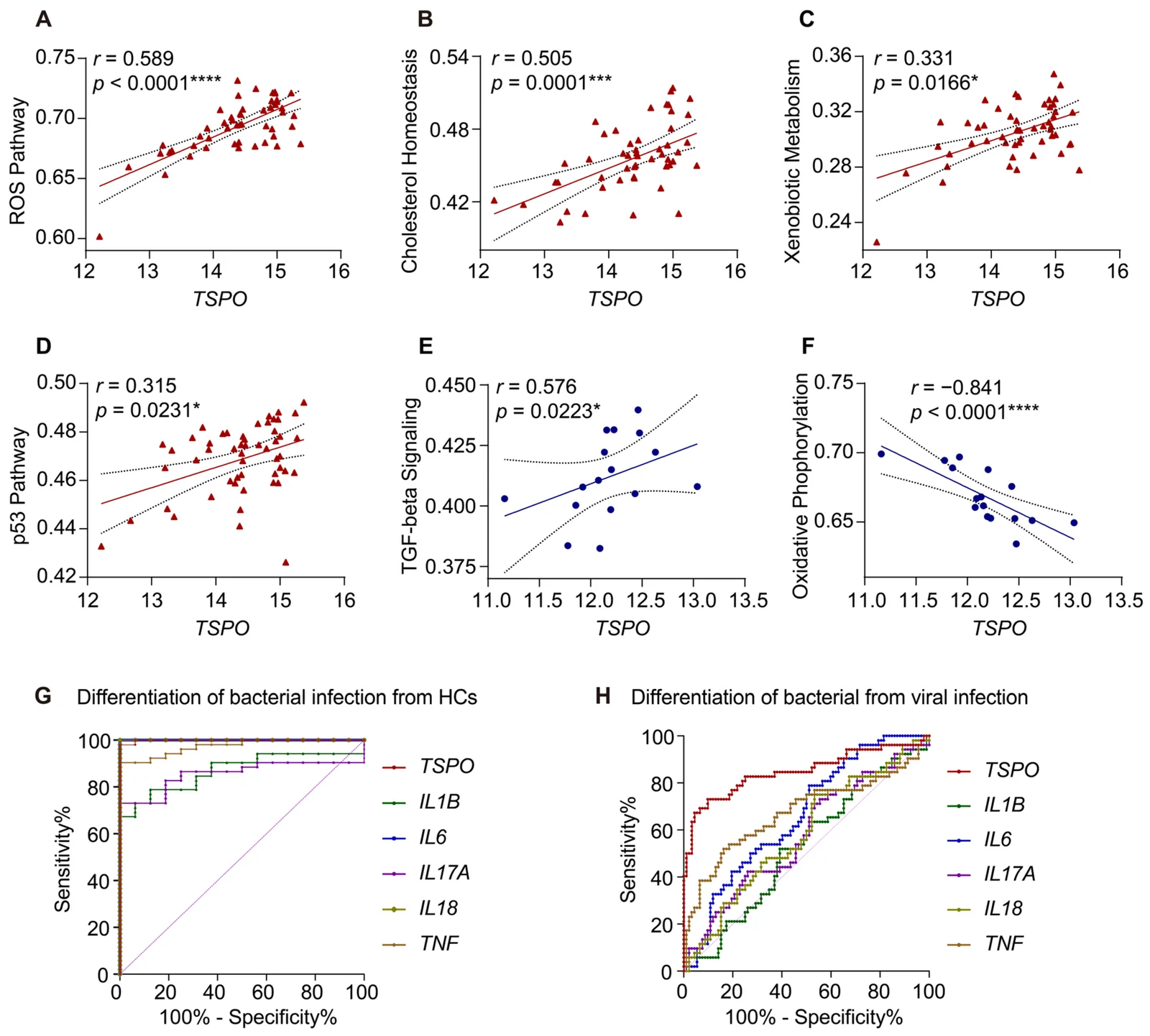
Correlations of *TSPO* expression with Hallmark pathways from MSigDB and diagnostic performance of TSPO. (**A**–**D**) Scatter plots showing Spearman rank correlation analyses between *TSPO* expression and ssGSEA enrichment scores of Hallmark pathways in definite bacterial infection group (red color, *n* = 52), including (**A**) reactive oxygen species (ROS) pathway, (**B**) cholesterol homeostasis, (**C**) xenobiotic metabolism, (**D**) p53 pathway. (**E**,**F**) Scatter plots showing Spearman rank correlation analyses between *TSPO* expression and ssGSEA scores of Hallmark pathways in HC group (blue color, *n* = 16), including (**E**) TGF-β signaling and (**F**) oxidative phosphorylation. (**G**,**H**) Receiver operating characteristic (ROC) curves comparing the diagnostic performance of *TSPO*, *IL1B*, *IL6*, *IL17A*, *IL18*, and *TNF* in (**G**) distinguishing bacterial from HCs, and (**H**) distinguishing bacterial from viral infection. Transcriptomic data were derived from the GSE72829 dataset. MSigDB, Molecular Signatures Database; ssGSEA, single-sample gene set enrichment analysis. * *p* < 0.05; *** *p* < 0.001; **** *p* < 0.0001.

To investigate the association between *TSPO* expression and infection-related transcriptional programs, Spearman correlation analyses were performed between *TSPO* mRNA levels and ssGSEA-derived Hallmark pathway scores in the bacterial infection and HC groups. In patients with bacterial infection, *TSPO* expression exhibited significant positive correlations with multiple metabolic and stress-response pathways (Figure 3A–D), including the ROS pathway (*r* = 0.589, *p* < 0.0001), cholesterol homeostasis (*r* = 0.505, *p* = 0.0001), xenobiotic metabolism (*r* = 0.331, *p* = 0.0166), and p53 pathway (*r* = 0.315, *p* = 0.0231). These findings indicated that *TSPO* upregulation during bacterial infection was closely associated with compensatory metabolic adaptation and stress-responsive transcriptional remodeling. In HCs (Figure 3E,F), *TSPO* expression was positively associated with TGF-β signaling (*r* = 0.576, *p* = 0.0223) and negatively correlated with oxidative phosphorylation (*r* = −0.841, *p* < 0.0001). These differences indicated that *TSPO* was linked to context-dependent metabolic and regulatory states, with its transcriptional associations being markedly reshaped during bacterial infection.

We further evaluated the association between *TSPO* expression and inflammatory pathway signatures in bacterial infection patients (Figure S2C). *TSPO* expression showed strong positive correlations with inflammatory response (*r* = 0.529, *p* < 0.0001), IL-6/JAK/STAT3 signaling (*r* = 0.697, *p* < 0.0001), and TNF-α signaling via NF-κB (*r* = 0.456, *p* = 0.0007). Collectively, these data demonstrated that *TSPO* expression was closely associated with coordinated activation of inflammatory, metabolic, and stress-response transcriptional programs during bacterial infection.

### 2.6. ROC Analysis Revealed Superior Diagnostic Performance of TSPO Compared with Conventional Inflammatory Cytokines

Receiver operating characteristic (ROC) curve analysis combined with DeLong’s test was performed to systematically compare the diagnostic capacity of *TSPO* and canonical inflammatory genes in two clinical discrimination scenarios: bacterial infection versus HCs, and bacterial infection versus viral infection (Table S3). For differentiating bacterial infection from HCs (Figure 3G), *TSPO* exhibited an excellent performance (AUC = 0.999, 95% CI: 0.995−1.000). *IL6* and *IL18* achieved perfect discrimination (AUC = 1.000 for both), while *TNF* (AUC = 0.974, 95% CI: 0.943−1.000), *IL1B* (AUC = 0.870, 95% CI: 0.787−0.954), and *IL17A* (AUC = 0.856, 95% CI: 0.767−0.945) exhibited relatively weaker predictive performance. DeLong’s test confirmed that *TSPO* achieved significantly higher AUC values than *IL1B* and *IL17A* (both *p* < 0.01), while no statistical differences were detected between *TSPO* and *IL6*, *IL18* or *TNF* (all *p* > 0.05), indicating that *TSPO* performed comparably to the strongest inflammatory cytokine markers in discriminating bacterial infection from HCs.

In the differentiation of bacterial versus viral infection (Figure 3H), *TSPO* remained robust diagnostic efficacy (AUC = 0.849, 95% CI: 0.774−0.924, *p* < 0.0001) at its optimal cut-off (>12.090). In contrast, *TNF* (AUC = 0.674) and *IL6* (AUC = 0.662) showed moderate discriminatory ability, whereas *IL1B*, *IL17A* and *IL18* failed to reach statistical significance (all *p* > 0.05). Importantly, DeLong’s test further verified that the AUC of *TSPO* was markedly higher than the other evaluated inflammatory cytokines in discriminating bacterial from viral infection (all *p* < 0.001), highlighting its potential as a superior diagnostic biomarker.

### 2.7. TLR4-Related Inflammatory Pathways Were Enriched, Whereas Calcium Signaling Was Transcriptionally Downregulated in Bacterial Infection

**Figure 4:**
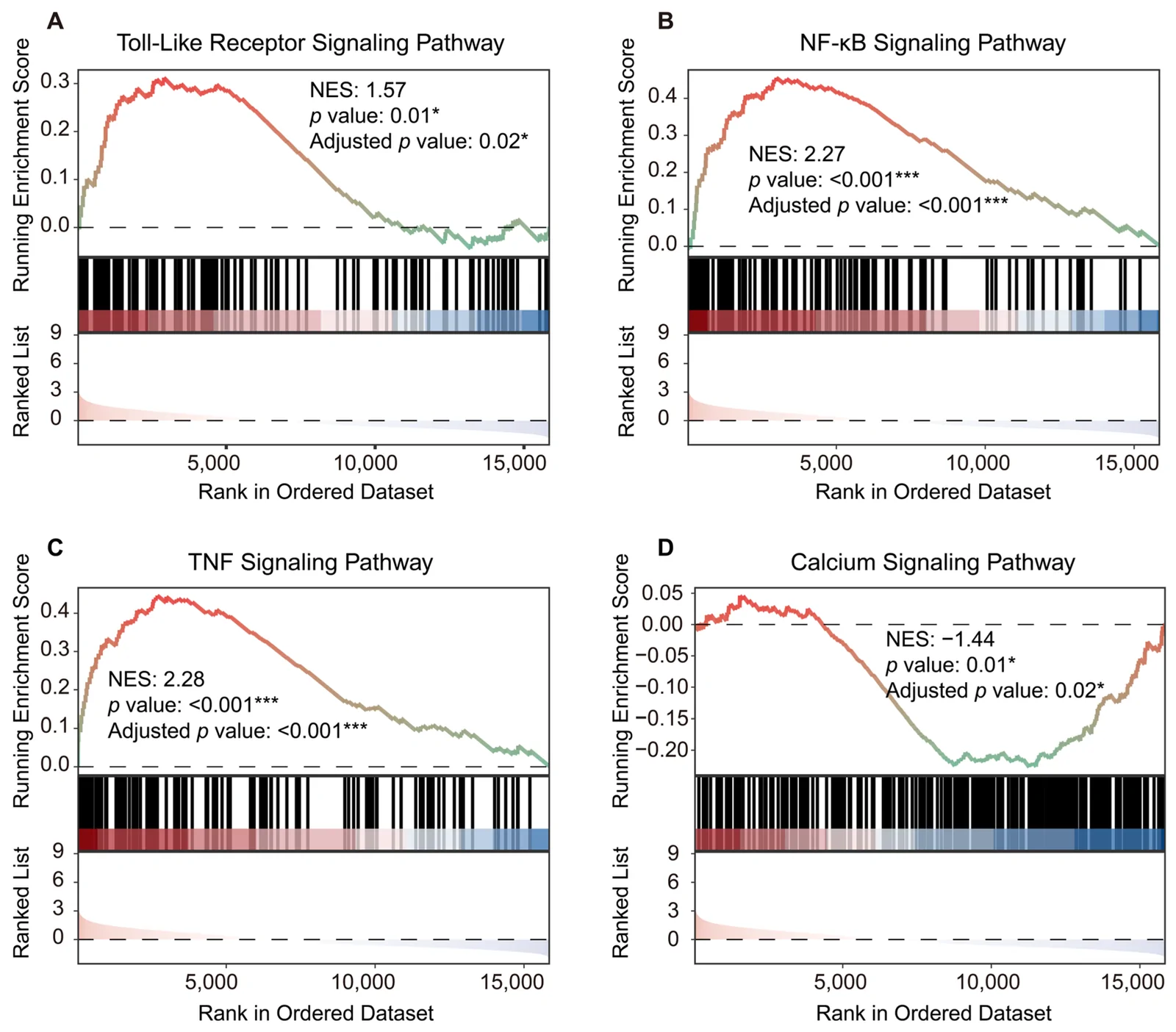
Enrichment of TLR4-related inflammatory cascades and negative enrichment of calcium signaling pathway in bacterial infection. (**A**–**D**) Gene Set Enrichment Analysis (GSEA) demonstrating the enrichment profiles of selected Kyoto Encyclopedia of Genes and Genomes (KEGG) pathways in patients with bacterial infection compared with HCs. Enrichment plots displayed the enrichment score (green-to-red curve) across the ranked gene list (x axis, rank in ordered dataset), with vertical black bars indicating the distribution of pathway-associated genes. Significant positive enrichment was observed for (**A**) Toll-like receptor signaling pathway, (**B**) NF-κB signaling pathway, and (**C**) TNF signaling pathway, whereas (**D**) the calcium signaling pathway exhibited significant negative enrichment. Data were retrieved from GSE72829. * *p* < 0.05; *** *p* < 0.001.

As shown in Figure 4, Gene Set Enrichment Analysis (GSEA) based on Kyoto Encyclopedia of Genes and Genomes (KEGG) pathway enrichment analysis revealed that the TLR signaling pathway was significantly enriched in patients with bacterial infection compared with HCs (NES = 1.57, adjusted *p* = 0.017). Consistently, two major downstream inflammatory cascades of TLR4 signaling, the NF-κB signaling pathway (NES = 2.27) and TNF signaling pathway (NES = 2.28), exhibited strong positive enrichment (both adjusted *p* < 0.001), indicating the enhanced activation of TLR4-driven inflammatory responses during bacterial infection. In contrast, the calcium signaling pathway exhibited significant negative enrichment (NES = −1.44, adjusted *p* = 0.019), suggesting a transcriptomic suppression of calcium homeostasis. Together, these findings indicated a coordinated molecular signature characterized by activation of TLR4-dependent inflammatory pathways accompanied by suppression of calcium signaling-related transcriptional responses in bacterial infection.

### 2.8. Murine TSPO Selectively Interacted with TLR4 and Is Enhanced by LPS Stimulation

**Figure 5:**
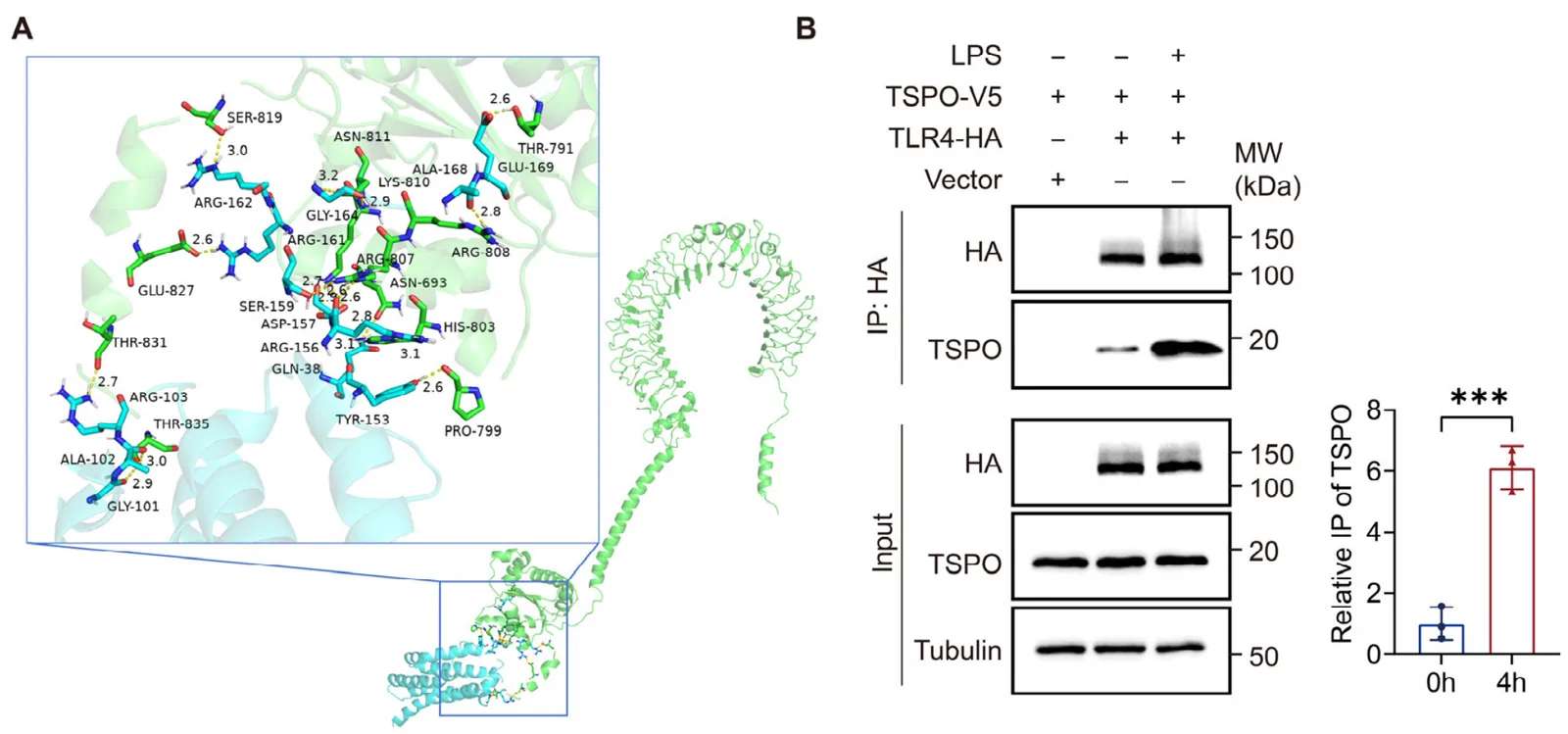
TSPO physically interacted with TLR4 and the interaction was enhanced by LPS stimulation. (**A**) Molecular docking prediction of the interaction interface between murine TSPO (blue ribbon) and TLR4 (green ribbon). The enlarged view (boxed region) highlighted the amino acid residues (rod-shaped structure) involved in hydrogen bonding (yellow dotted lines) that stabilized the binding interface. (**B**) Co-immunoprecipitation (Co-IP) validation of the TLR4-TSPO interaction in HEK293T cells. Input samples confirmed comparable expression levels of transfected proteins, and no interaction was detected in empty-vector controls. Right bar showing the quantification of the relative IP efficiency of TSPO-V5 as normalized to TLR4-HA expression at baseline and 4 h after stimulation with LPS (1 μg/mL). Data were presented with three independent experiments. *** *p* < 0.001.

To investigate the potential molecular interaction between TSPO and TLR4, molecular docking analysis was performed using the predicted structures of murine TSPO and TLR4. As shown in Figure 5A, the C-terminal region of TSPO was predicted to interact with the Toll/interleukin-1 receptor (TIR) domain of TLR4, forming a stable binding interface. LigPlot+ analysis (Figure S3A) further revealed that multiple amino acid residues contributed to the interaction through hydrogen bonding and hydrophobic interactions, supporting the potential formation of a TLR4-TSPO complex.

To experimentally validate this predicted interaction, HEK293T cells were co-transfected with plasmids encoding TLR4-HA and TSPO-V5, followed by co-immunoprecipitation (Co-IP) analysis. TSPO was readily detected in TLR4-HA immunoprecipitates, confirming the association between TSPO and TLR4 (Figure 5B). Notably, LPS stimulation significantly enhanced the interaction between TSPO and TLR4 (*p* < 0.001), suggesting that inflammatory activation promoted the formation of the TLR4-TSPO complex. In contrast, no detectable interaction was observed between TSPO and other endosomal TLRs, including TLR7 and TLR9 (Figure S3B,C), indicating that TSPO selectively associated with TLR4 among the tested TLR family members.

### 2.9. Cell-Type-Specific Expression Profiles of TLR4 and TSPO in Brain Tissue and Peripheral Blood

To identify the major cellular populations expressing *TLR4* and *TSPO* in the central nervous system (CNS) and peripheral circulation, we performed cell-type-resolved transcriptomic analysis using the CZ CELLxGENE Discover platform [14]. In healthy human brain tissue (Figure S4A,B), both *TLR4* and *TSPO* transcripts showed preferential enrichment in microglia compared with other major brain cell populations. Consistently, *Tlr4* and *Tspo* also showed enriched expression in microglial cells across mouse brain cell populations (Figure S4C,D), though not at the maximal level. We further investigated the expression pattern of *TSPO* in peripheral blood-derived cell populations (Figure S4E,F). In both human and mouse blood transcriptomic datasets, *TSPO* showed preferential enrichment in macrophage populations. Collectively, these cell-type-specific expression profiles identified microglia and macrophages as major cellular populations associated with *TLR4* and *TSPO* expression, providing a cellular basis for further investigation of their immunomodulatory roles during inflammatory responses.

### 2.10. Conserved Co-Expression of TSPO with TLR4 Signaling and Anti-Inflammatory Regulatory Genes in Human and Murine Brain Cells

To investigate the potential immunoregulatory role of *TSPO* in the CNS, Spearman correlation analyses were performed to assess the associations between *TSPO* expression and key immune regulatory genes across human and murine brain single-cell transcriptomic datasets derived from the CELLxGENE platform (Figure S5). In human brain cell populations (Figure S5A–D), *TSPO* expression exhibited significant positive correlations with *TLR4* (*r* = 0.344, *p* = 0.0002), *HMOX1* (*r* = 0.224, *p* = 0.0047), *ARG1* (*r* = 0.577, *p* < 0.0001), and *IL10* (*r* = 0.524, *p* < 0.0001). Consistently, analysis of murine brain single-cell transcriptomes demonstrated that *Tspo* was positively correlated with the corresponding homologous genes, including *Tlr4* (*r* = 0.287, *p* = 0.0056), *Hmox1* (*r* = 0.631, *p* < 0.0001), *Arg1* (*r* = 0.720, *p* < 0.0001), and *Il10* (*r* = 0.666, *p* < 0.0001) (Figure S5E–H). The conserved co-expression patterns observed across human and mouse brain cells indicated that *TSPO* was transcriptionally coupled with *TLR4*-associated signaling as well as *HMOX1*-, *ARG1*-, and *IL10*-mediated anti-inflammatory programs. These findings suggested that TSPO may participate in maintaining immune–metabolic homeostasis in CNS-resident cells through coordinated regulation of inflammatory activation and compensatory protective responses.

### 2.11. Tspo Knockout Enhanced LPS-Induced Inflammatory Responses in BV2 Microglia

**Figure 6:**
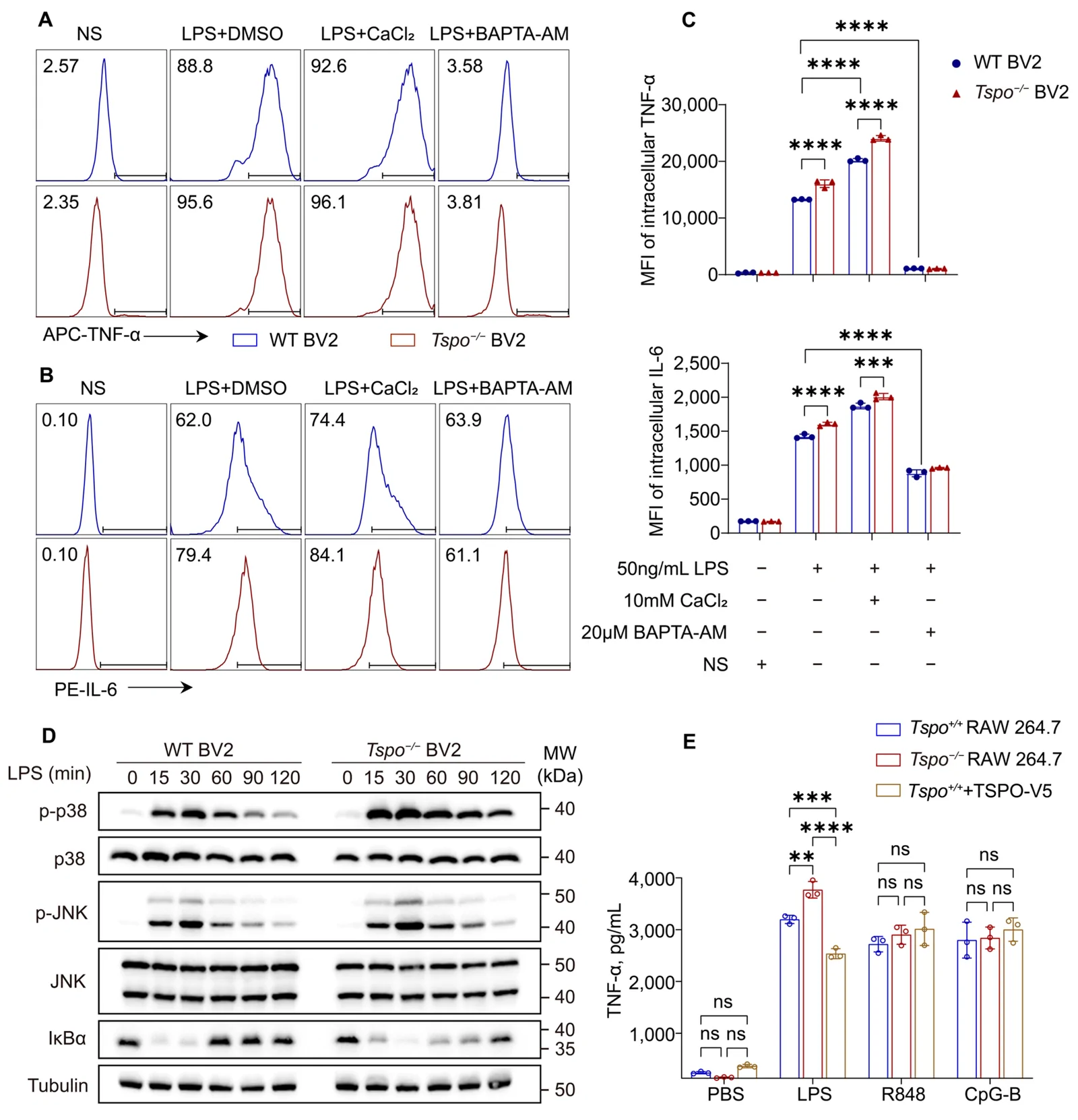
TSPO deficiency selectively enhanced LPS-induced pro-inflammatory responses through MAPK and NF-κB pathways. (**A**–**C**) Intracellular cytokine staining (ICS) analysis of intracellular (**A**) TNF-α and (**B**) IL-6 levels in WT and *Tspo*^−/−^ BV2 cells. (**C**) Right panels were the quantitation of median fluorescence intensity (MFI). *Tspo*^−/−^ BV2 cells (red) exhibited significantly higher TNF-α and IL-6 levels than WT cells (blue) upon LPS stimulation and CaCl_2_ supplementation, while BAPTA-AM attenuated the production of pro-inflammatory cytokines in both cell types. (**D**) Western blot analysis demonstrating the phosphorylation of p38 and JNK, as well as the degradation of IκBα were more markedly in *Tspo*^−/−^ than WT BV2 cells following 50 ng/mL LPS stimulation, indicating the enhanced activation of MAPK and NF-κB signaling pathways in TSPO deficiency. Tubulin served as a loading control. (**E**) ELISA quantification of secreted TNF-α in culture supernatants from *Tspo*^+/+^, *Tspo*^−/−^, and *Tspo*^+/+^+TSPO-V5 RAW 264.7 cells. Cells were treated with PBS, the TLR4 agonist LPS (100 ng/mL, 1 h), TLR7 agonist R848 (15 ng/mL, 6 h), or TLR9 agonist CpG-B (200 nM, 6 h). All experiments were performed in at least three independent biological replicates. ** *p* < 0.01; *** *p* < 0.001; **** *p* < 0.0001; ns, not significant.

*Tspo*^−/−^ BV2 microglia cell lines were generated using the CRISPR-Cas9 gene-editing system (Figure S6A). As presented in Figure 6A–C, under basal unstimulated (NS) conditions, the pro-inflammatory cytokines TNF-α and IL-6 were barely detectable in both WT and *Tspo*^−/−^ BV2 cells. Following stimulation with 50 ng/mL LPS for 4 h, robust upregulation of TNF-α and IL-6 was observed in both groups. Notably, *Tspo*^−/−^ BV2 cells exhibited markedly higher cytokine levels compared with WT controls (both *p* < 0.0001). These results indicated that TSPO deficiency significantly potentiated LPS-induced inflammatory responses in BV2 microglia.

### 2.12. Calcium Signaling Contributed to LPS-Induced Inflammatory Responses in BV2 Cells

To assess the role of Ca^2+^ in inflammation, extracellular Ca^2+^ was supplemented using CaCl_2_ together with 50 ng/mL LPS stimulation, which further increased the secretion of TNF-α and IL-6 in both WT and *Tspo*^−/−^ BV2 cells, as compared to the simple LPS stimulation (Figure 6A–C). In contrast, treatment with the Ca^2+^ chelator BAPTA-AM significantly reduced the levels of TNF-α and IL-6 across both cell lines. These findings suggested that Ca^2+^ signaling acted as a positive regulator in LPS-induced inflammatory responses.

### 2.13. TSPO Deficiency Enhanced the Activation of MAPK and NF-κB Pathways in BV2 Cells

Western blot analysis was performed to characterize the activation status of MAPK and NF-κB signaling axes (Figure 6D). Under basal conditions, total protein abundances of p38 and JNK showed comparable levels between WT and *Tspo*^−/−^ BV2 cells. Upon 50 ng/mL LPS stimulation, phosphorylation of p38 and JNK increased in a time-dependent manner in both cell groups. Compared with WT cells, *Tspo*^−/−^ BV2 cells exhibited significantly elevated phosphorylation of p38 and JNK, suggesting the enhanced activation of the MAPK signaling pathway. In parallel, IκBα degradation was more pronounced in *Tspo*^−/−^ BV2 cells than WT cells, indicating the enhanced activation of the NF-κB signaling pathway in TSPO deficiency.

### 2.14. TSPO Selectively Suppressed TLR4-Mediated Inflammatory Responses upon LPS Stimulation

To further investigate whether TSPO selectively regulated TLR4 pathways, we generated *Tspo*^−/−^ RAW 264.7 macrophages using CRISPR-Cas9 editing (Figure S6B) and generated the overexpressed cell line by lentiviral delivery of V5-tagged TSPO into *Tspo*^+/+^ RAW 264.7 cells (*Tspo*^+/+^+TSPO-V5, Figure S6C). Three groups of cells were stimulated with ligands targeting distinct TLR pathways, including TLR4 agonist LPS (100 ng/mL, 1 h), TLR7 agonist R848 (15 ng/mL, 6 h), and TLR9 agonist CpG-B (200 nM, 6 h), and supernatant TNF-α concentrations were quantified by ELISA (Figure 6E). Upon LPS stimulation, *Tspo*^−/−^ cells exhibited significantly higher TNF-α production compared to *Tspo*^+/+^ controls (*p* < 0.01), whereas *Tspo*^+/+^+TSPO-V5 cells drastically reduced LPS-triggered TNF-α release relative to both *Tspo*^−/−^ and *Tspo*^+/+^ cells (*p* < 0.001). In contrast, TSPO deletion or overexpression had no significant effects on TNF-α production following R848 or CpG-B stimulation. These findings demonstrated that TSPO selectively restrained TLR4-mediated inflammatory activation without affecting TLR7- or TLR9-dependent cytokine responses.

### 2.15. TSPO Deficiency Remodeled Intracellular Ca^2+^ Dynamics During Inflammatory Activation

**Figure 7:**
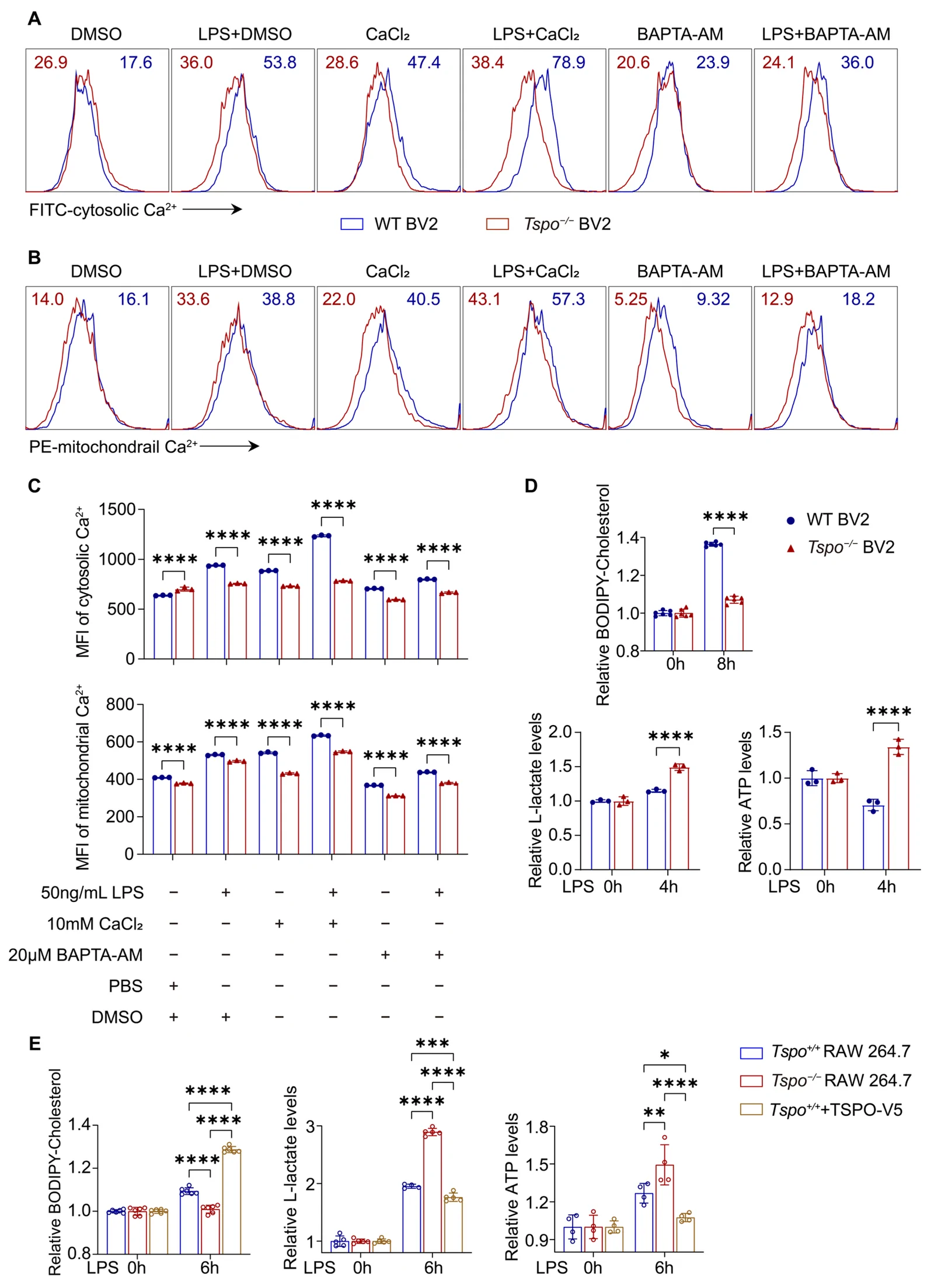
TSPO deficiency disrupted Ca^2+^ homeostasis and Induced Compensatory Metabolic Remodeling During Inflammatory Stress. (**A**,**B**) Flow cytometric analysis of intracellular Ca^2+^ levels in WT (blue) and *Tspo*^−/−^ (red) BV2 cells. Representative histograms showed (**A**) FITC-cytosolic Ca^2+^ and (**B**) PE-mitochondrial Ca^2+^ signals. (**C**) Quantification of MFI for cytosolic (top) and mitochondrial (bottom) Ca^2+^ levels. *Tspo*^−/−^ cells exhibited significantly reduced mitochondrial Ca^2+^ uptake under basal and stimulated conditions, coupled with the impaired cytosolic Ca^2+^ responses to LPS and CaCl_2_ supplementation as compared to WT cells. BAPTA-AM treatment reduced both cytosolic and mitochondrial Ca^2+^ levels. (**D**) Bar graphs showing relative cholesterol, L-lactate and ATP levels in WT and *Tspo*^−/−^ BV2 cells before and upon LPS stimulation (50 ng/mL). (**E**) Bar graphs showing relative cholesterol levels, relative L-lactate concentrations, and relative ATP abundance in *Tspo*^+/+^, *Tspo*^−/−^, and *Tspo*^+/+^+TSPO-V5 RAW 264.7 macrophages before (0 h) and after 6 h of LPS stimulation (100 ng/mL). All experiments were performed in at least three independent biological replicates. * *p* < 0.05; ** *p* < 0.01; *** *p* < 0.001; **** *p* < 0.0001.

As shown in Figure 7A–C, under basal conditions, *Tspo*^−/−^ BV2 cells exhibited a modest but significant increase in cytosolic Ca^2+^ levels accompanied by a marked reduction in mitochondrial Ca^2+^ levels compared with WT cells (both *p* < 0.0001), suggesting altered intracellular Ca^2+^ distribution and impaired mitochondrial Ca^2+^ handling following TSPO deletion. Following stimulation with 50 ng/mL LPS, WT cells displayed robust elevations in both cytosolic and mitochondrial Ca^2+^ levels. In contrast, *Tspo*^−/−^ cells exhibited significantly attenuated Ca^2+^ responses compared with WT cells upon LPS stimulation, indicating that TSPO deficiency impaired Ca^2+^ dynamics during inflammatory activation. Upon extracellular Ca^2+^ supplementation with CaCl_2_, WT cells showed pronounced increases in both cytosolic and mitochondrial Ca^2+^ levels, whereas *Tspo*^−/−^ cells displayed substantially blunted Ca^2+^ elevations. Conversely, treatment with the intracellular Ca^2+^ chelator BAPTA-AM markedly reduced cytosolic and mitochondrial Ca^2+^ signals in both WT and *Tspo*^−/−^ cells. Collectively, these findings indicated that TSPO deficiency disrupted intracellular Ca^2+^ homeostasis and remodeled Ca^2+^ handling responses under inflammatory stress.

### 2.16. TSPO-Regulated Cholesterol Handling and Metabolic Adaptation During Inflammatory Stress

Accumulating evidence implicated TSPO in mitochondrial lipid transport and cholesterol metabolism [15]; we therefore investigated whether TSPO deficiency perturbed cellular cholesterol homeostasis. Intracellular cholesterol levels were assessed using Boron–Dipyrromethene (BODIPY)–cholesterol fluorescence in WT and *Tspo*^−/−^ BV2 cells, alongside *Tspo*^+/+^, *Tspo*^−/−^, and *Tspo*^+/+^+TSPO-V5 RAW 264.7 macrophages. As shown in Figure 7D, E and Figure S7, *Tspo*^−/−^ BV2 cells displayed significantly reduced BODIPY–cholesterol fluorescence intensity relative to WT cells (*p* < 0.0001), demonstrating impaired intracellular cholesterol accumulation in the absence of TSPO. Consistently, *Tspo*^−/−^ RAW 264.7 macrophages showed decreased BODIPY–cholesterol intensity, whereas *Tspo*^+/+^+TSPO-V5 cells markedly increased intracellular cholesterol accumulation, with WT cells displaying an intermediate phenotype (all *p* < 0.0001). These findings indicated that TSPO positively regulated cellular cholesterol handling and contributed to the maintenance of lipid metabolic homeostasis under inflammatory conditions.

We next examined whether TSPO deficiency altered cellular metabolic adaptation following inflammatory challenge. Upon exposure to 50 ng/mL LPS, intracellular L-lactate levels were significantly increased in both WT and *Tspo*^−/−^ BV2 cells. Notably, *Tspo*^−/−^ BV2 cells exhibited substantially higher L-lactate accumulation than WT cells upon LPS stimulation (*p* < 0.0001), suggesting amplified lactate production and metabolic adaptation in the absence of TSPO. To further evaluate the impact of TSPO deficiency on cellular energy maintenance, intracellular ATP levels were measured during inflammation. As results, *Tspo*^−/−^ BV2 cells maintained significantly higher ATP levels compared with WT controls following LPS stimulation (Figure 7D), suggesting preserved cellular energy availability despite increased inflammatory activation.

To determine whether these metabolic alterations were dependent on TSPO levels, we further performed parallel experiments in *Tspo*^+/+^, *Tspo*^−/−^, and *Tspo*^+/+^+TSPO-V5 RAW 264.7 macrophages. Consistent with the findings in BV2 microglia, *Tspo*^−/−^ RAW 264.7 cells exhibited increased L-lactate accumulation and sustained ATP levels following LPS stimulation. In comparison, *Tspo*^+/+^+TSPO-V5 cells displayed reduced L-lactate production and attenuated ATP preservation compared with WT cells (Figure 7E). Collectively, these results indicated that TSPO levels modulated metabolic adaptation during inflammatory conditions, with TSPO deficiency promoting a compensatory metabolic state characterized by impaired cholesterol accumulation, increased L-lactate production, and maintained energy availability.

### 2.17. Enrichment Analysis Revealed Coordinated Activation of TLR-Associated Immune and Immunometabolic Programs

To characterize transcriptional programs associated with *TSPO* expression during bacterial infection, we performed KEGG and Gene Ontology (GO) enrichment analyses of genes significantly correlated with *TSPO* expression using transcriptomic datasets from patients with bacterial infection. KEGG enrichment analysis revealed significant enrichment of the TLR signaling pathway, together with immunometabolic pathways including hypoxia-inducible factor 1 (HIF-1) signaling (Figure S8A). In addition, pathways involved in lysosomal function, phagosome formation, and endocytosis were significantly enriched, suggesting that TSPO-associated transcriptional programs were linked to innate immune activation, phagocytic processes, and metabolic regulation during bacterial infection.

Consistent with the KEGG analysis, GO enrichment analysis further demonstrated that TSPO-correlated genes were enriched in immune-related and metabolic processes (Figure S8B). Biological Process (BP) analysis identified enrichment of terms associated with leukocyte activation, cytokine production, and innate immune responses, indicating an association between *TSPO* expression and inflammatory immune programs. Cellular Component (CC) analysis revealed enrichment of phagosomes, lysosomes, cytoplasmic vesicles, and secretory granules. Molecular Function (MF) analysis showed enrichment of immune receptor activity and TLR-associated molecular functions. Collectively, these transcriptomic analyses demonstrated that *TSPO* expression was associated with coordinated activation of TLR-related immune signatures and immunometabolic pathways during bacterial infection.

## 3. Discussion

In the present study, we combined transcriptomic analysis with in vitro functional experiments to investigate the role of TSPO in the TLR4 signaling pathway. Our data consistently identified TSPO as a potential modulator that integrated inflammatory signaling with immunometabolic adaptation during inflammatory conditions, which was functionally associated with TLR4 signaling activation, intracellular Ca^2+^ homeostasis, cholesterol metabolism, and cellular energy adaptation. Importantly, our findings highlighted the clinical value of TSPO as both a diagnostic biomarker and a mechanistic node in bacterial infection.

### 3.1. TSPO Selectively Associates with TLR4 to Modulate Inflammatory Signaling

Clinical transcriptomic data and Co-IP assays corroborated the preferential TSPO-TLR4 association, indicating that the immunoregulatory role of TSPO was pathway-specific, rather than a general suppressor of innate immunity. Functionally, TSPO deficiency amplified LPS-induced cytokine production, whereas TSPO restoration restrained excessive inflammatory activation, suggesting that endogenous TSPO served as a buffering mechanism to maintain immune homeostasis during TLR4 activation [16,17]. Although our data established an association between TSPO and TLR4-mediated inflammation, the precise molecular mechanism by which TSPO regulated TLR4 activation remained to be determined. Future studies should clarify whether TSPO affects TLR4 trafficking, adaptor recruitment, or downstream signal propagation.

### 3.2. TSPO Integrates TLR4-Mediated Inflammation with Immunometabolic Adaptation

Beyond its involvement in TLR4 signaling, our findings suggested that TSPO functioned as a regulator that coordinated inflammatory activation with metabolic adaptation. Effective inflammatory responses required the balance between pathogen clearance and the prevention of excessive inflammatory damage [18]. In this context, elevated *TSPO* expression during bacterial infection may represent an endogenous adaptive mechanism that counteracted inflammatory stress and maintained cellular homeostasis, potentially through the integration of mitochondrial function and metabolic regulation [19,20]. Mechanistically, the coupling between TSPO-associated inflammatory and metabolic programs provided a potential explanation for its regulatory role in TLR4 signaling. Mitochondria served as central platforms that coupled innate immune activation with cellular metabolism, thereby determining the threshold and duration of inflammatory responses [21]. Thus, TSPO may act as a molecular bridge that coordinated TLR4 activation with metabolic pathways, enabling immune cells to adapt to inflammatory demands while preventing excessive cytokine production. Collectively, these findings expanded the current concept of TSPO from an inflammation-associated biomarker to an active regulator of immunometabolic homeostasis during inflammatory stress.

### 3.3. TSPO Maintains Calcium Homeostasis to Modulate TLR4-Mediated Inflammatory Responses

Our findings indicated that TSPO served as an important regulator of cellular calcium homeostasis, thereby influencing the magnitude of TLR4-driven inflammatory activation. Consistently, previous studies have suggested that the knockdown of TSPO in BV2 cells significantly enhanced LPS-induced TNF-α expression and secretion [22]. Furthermore, pharmacological activation of TSPO using 4′-Chlorodiazepam (Ro5-4864) was reported to upregulate the *TSPO* expression and reduce the release of pro-inflammatory cytokines [23]. These results suggested that TSPO was able to suppress the intensity of inflammatory responses, beyond its conventional role in mitochondrial cholesterol transport.

In addition, *Tspo*^−/−^ BV2 cells exhibited increased cytosolic Ca^2+^ levels accompanied by reduced mitochondrial Ca^2+^ levels under baseline conditions, which may be attributed to the impaired mitochondrial membrane potential following TSPO ablation, leading to reduced mitochondrial Ca^2+^ uptake and storage capacity [24]. As a result, Ca^2+^ failed to efficiently enter the mitochondrial matrix and instead accumulated in the cytosol [24,25].

Given the role of mitochondrial Ca^2+^ in regulating metabolic enzymes and oxidative phosphorylation, the impairment of the mitochondrial Ca^2+^ uptake was likely to compromise oxidative metabolism [26]. However, despite reduced Ca^2+^ responsiveness upon LPS stimulation, *Tspo*^−/−^ BV2 cells exhibited enhanced inflammatory responses compared with WT cells. This seemingly paradoxical phenomenon suggested that the role of Ca^2+^ in inflammation was not solely determined by its absolute levels, but rather depended on its subcellular distribution and dynamic flux [27]. Mechanistically, TSPO deficiency may weaken the mitochondrial Ca^2+^ buffering, leading to the dysregulated Ca^2+^ microdomains, which could potentiate the aberrant activation of downstream inflammatory pathways [28]. In addition, TSPO deficiency has been associated with the enhanced autophagy and ROS production [29,30], which could further facilitate the activation of pro-inflammatory pathways that were modulated by both Ca^2+^ signaling and redox status, such as NF-κB and inflammasome activation [31,32].

### 3.4. TSPO Functions to Maintain Immunometabolic Homeostasis During Inflammatory Stress

Our findings suggested that TSPO served as an important component that coordinated metabolic adaptation during inflammatory activation, which may function to integrate cholesterol trafficking, energy production, and inflammatory signaling to preserve cellular homeostasis. Upon inflammatory stimulation, immune cells required metabolic adaptation to support increased biosynthetic demands, signaling transduction, and energy consumption [33,34]. In this context, the metabolic phenotype associated with TSPO deficiency may represent a compensatory adaptation that allowed cells to maintain energetic capacity under inflammatory stress. The enhanced glycolytic tendency in TSPO-deficient cells resembled features of the metabolic phenotype commonly observed in activated immune cells [35,36], where increased glycolysis provided rapid ATP generation and supplied metabolic intermediates required for inflammatory responses [36,37,38]. However, whether TSPO deficiency truly induced metabolic reprogramming, or instead promoted adaptive metabolic shifts secondary to altered mitochondrial homeostasis, remained to be clarified. Future studies incorporating measurements of glycolytic flux, mitochondrial respiration, and glucose utilization will be required to further determine how TSPO influences cellular metabolism during inflammatory activation.

### 3.5. Clinical Significance of TSPO as a Potential Biomarker for Bacterial Infection

Beyond its mechanistic role in immune regulation, our findings highlight the potential clinical value of TSPO as a diagnostic biomarker for bacterial infection. Although cytokines such as IL-6 and IL-18 exhibited excellent discriminatory performance for distinguishing bacterial infection from HCs, their diagnostic utility was substantially reduced when differentiating bacterial from viral infections, likely due to their shared involvement in diverse inflammatory conditions. In contrast, TSPO retained a strong discriminatory capacity in distinguishing bacterial from viral infection, suggesting that it may provide additional information beyond general inflammatory activation. The diagnostic advantage of TSPO may be related to its selective association with TLR4 signaling, which was preferentially activated in bacterial infection. Therefore, TSPO may represent a molecular signature reflecting the specific infection induced by bacteria rather than simply the severity of inflammation. Given its established application as a molecular imaging marker of neuroinflammation [39], TSPO may have broader translational potential as a complementary biomarker to improve the identification and stratification of bacterial infections.

**Figure 8:**
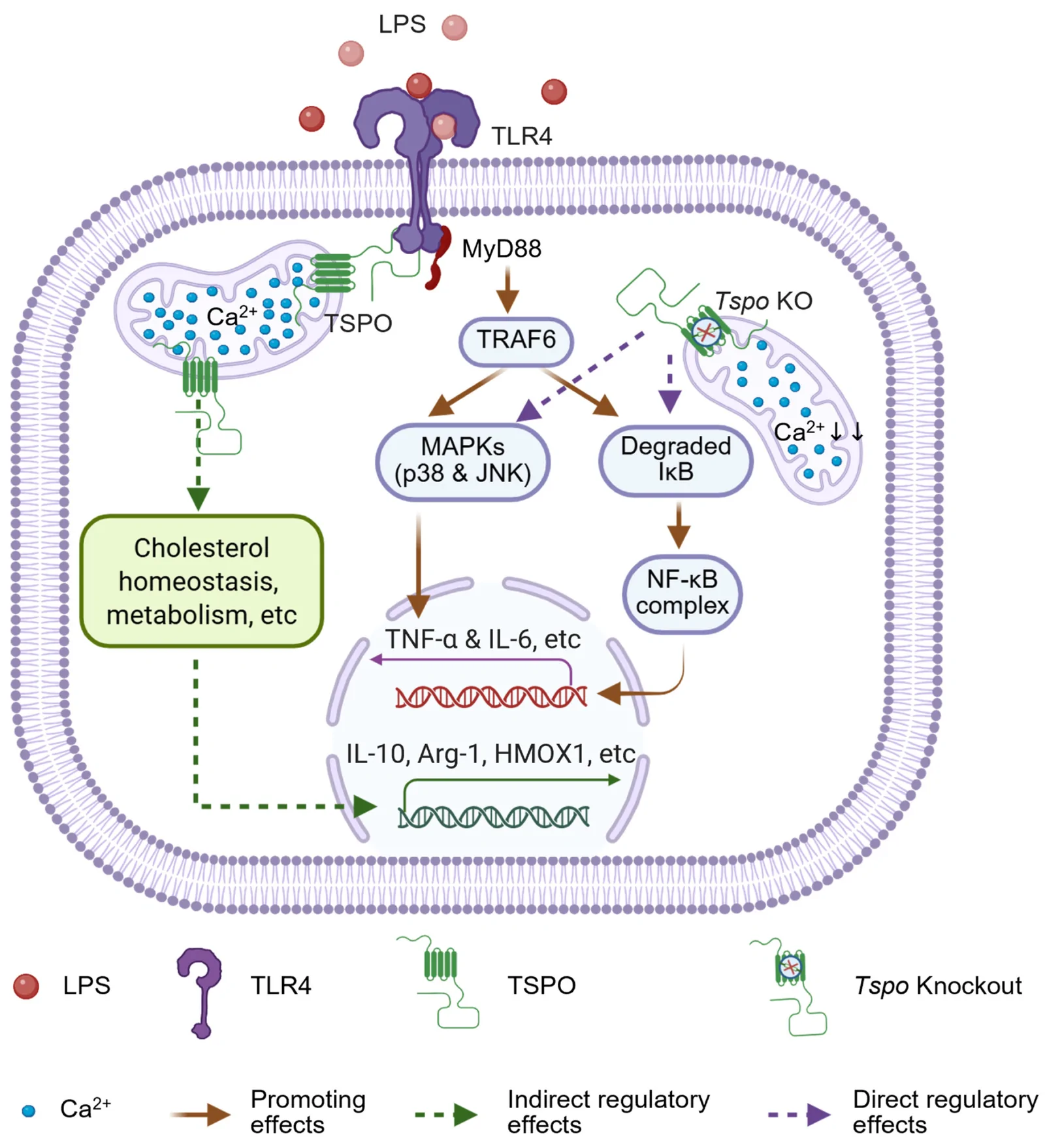
Schematic illustration of TSPO‑mediated negative regulation of TLR4 inflammatory signaling. Canonical TLR4 signaling was initiated by LPS stimulation and thus activated the downstream MAPK (p38 and JNK phosphorylation) and NF-κB (IκBα degradation) pathways to induce pro-inflammatory cytokines (TNF-α and IL-6). In WT cells, TSPO interacted with the TLR4 complex, maintained intracellular Ca^2+^ homeostasis, and indirectly promoted the production of anti-inflammatory cytokines (IL-10 and Arg-1) and antioxidant enzyme (Hmox1) through the anti-inflammatory pathways (cholesterol homeostasis and ROS pathways). In *Tspo*^−/−^ cells, TSPO deficiency disrupted mitochondrial Ca^2+^ uptake, and amplified the activation of MAPK and NF-κB pathways. In addition, the absence of TSPO led to the impaired oxidative metabolism and metabolic adaptation. The resulting excessive pro-inflammatory cytokine production exacerbated inflammatory responses, thus highlighting TSPO as a negative regulator of TLR4 signaling in innate immune cells. Created in BioRender. XQ Wu. (2026) https://BioRender.com/wj675ri.

Based on these findings, we proposed a mechanistic model (Figure 8), in which mitochondrial TSPO maintained intracellular Ca^2+^ homeostasis and supported cellular metabolism through cholesterol homeostasis, and ROS regulation, which promoted the expression of anti-inflammatory molecules (e.g., IL-10, Arg-1, and HMOX1). Genetic ablation of TSPO disrupted mitochondrial Ca^2+^ uptake, driven a shift toward compensatory metabolism adaptation, and enhanced the LPS-induced inflammatory responses through the excessive activation of MAPK and NF-κB pathways, which upregulated the transcription of pro-inflammatory cytokines (TNF-α and IL-6).

### 3.6. Limitations and Future Perspectives

Several limitations should be acknowledged in this study. Firstly, TSPO resides on the outer mitochondrial membrane, while TLR4 predominantly distributes at the plasma membrane and endosomal compartments. The subcellular microenvironment, protein membrane topology, and precise binding interface supporting TSPO-TLR4 association remain incompletely characterized. Secondly, this static measurement of Ca^2+^ levels cannot capture real-time Ca^2+^ flux, mitochondrial Ca^2+^ uptake kinetics, or local ion microdomain dynamics. Thus, high-resolution live-cell imaging techniques are needed to dissect dynamic Ca^2+^ signaling and clarify its causal relationship with downstream inflammatory pathways.

## 4. Materials and Methods

### 4.1. Transcriptomic Dataset Acquisition

The publicly available transcriptomic dataset GSE72829 was retrieved from the NCBI Gene Expression Omnibus (GEO) database [13]. In this study, we analyzed the RNA signatures of peripheral blood from patients with definite bacterial infection (*n* = 52), definite viral infection (*n* = 92), and HCs (*n* = 16) that derived from the dataset [13].

### 4.2. Functional and Pathway Enrichment Analysis

In this study, functional analyses of DEGs were performed using the limma package (version 3.60.4) with R software (version 4.6.1) to characterize transcriptional differences among patients with definite bacterial infection, definite viral infection, and HCs. For the comparison between bacterial infection and HCs, DEGs were defined using a stringent threshold of *p* < 1 × 10^−15^ and log_2_ fold change (|log_2_FC|) > 1. For the comparison between bacterial and viral infections, DEGs results with *p* < 1 × 10^−5^ and |log_2_FC| > 0.5 were considered statistically significant. Additionally, Gene Set Enrichment Analysis (GSEA) was performed to investigate the pathway differences between two groups using clusterProfiler package (version 4.20.0) in the R environment. GSEA mainly focused on KEGG pathways and GO terms.

### 4.3. Calculation of Gene Set Enrichment Scores of Hallmark Pathways and Correlation Analysis with TSPO Gene Expression

Single-sample Gene Set Enrichment Analysis (ssGSEA) was conducted using the GSVA software package (version 1.42.0) in R software to calculate the enrichment score (ES) of hallmark pathways derived from the Molecular Signatures Database (MSigDB) in both the definite bacterial infection group and the HC group, based on Transcripts Per Million (TPM)-normalized gene expression data. Subsequently, Spearman rank correlation analysis was carried out to explore the correlation between the ssGSEA-derived ES and TSPO gene expression levels.

### 4.4. Gene Correlation Analysis

Genes significantly correlated with *TSPO* expression were subjected to Gene Ontology (GO) and Kyoto Encyclopedia of Genes and Genomes (KEGG) enrichment analyses using the R package cluster Profiler. Gene symbols were converted to Entrez IDs via the org.Hs.eg.db annotation database. For GO enrichment, three ontology categories including biological process (BP), cellular component (CC), and molecular function (MF) were analyzed simultaneously. The Benjamini–Hochberg (BH) method was applied for multiple testing correction, with thresholds set as *p* value Cutoff = 0.05 and q value Cutoff = 0.05. KEGG enrichment was performed against the human pathway database with identical significance thresholds. The set Readable function was used to convert enrichment results back to gene symbols for intuitive interpretation. The top 10 enriched terms of each GO category and top 20 significant KEGG pathways were extracted, and Gene Ratio values were calculated. Visualization of enrichment results, including GO bar plots, GO bubble plots and KEGG bubble plots, was implemented with the ggplot2 package (version 4.0.3).

### 4.5. Single-Cell Transcriptomic Analysis

Single-cell transcriptomic datasets from healthy human and mouse brain tissues, as well as peripheral blood, were analyzed using the Chan Zuckerberg CELLxGENE Discover platform (https://cellxgene.cziscience.com/) [14] to characterize the expression patterns of TLR4 and TSPO across different cell populations. In addition, Spearman correlation analysis was performed to evaluate the associations between *TSPO* expression and the expression levels of *TLR4*, *HMOX1*, *ARG1*, and *IL10* within individual cell populations from healthy human and mouse brain tissues.

### 4.6. Cell Lines and Culture Conditions

BV2 cell line was a gift of Prof. Fang Huang [40], and routinely underwent short tandem repeat (STR) detection for cell line authentication as carried out by Cell Bank/Stem Cell Bank, SIBCB, CAS. HEK293T and RAW 264.7 macrophage cell lines were kindly provided by Prof. Bo Liu [41]. Cells were grown and maintained in Dulbecco’s modified Eagle medium (DMEM, C11995500CP, Gibco, Waltham, MA, USA) that was supplemented with 10% fetal bovine serum (FBS, 40130ES76, Yeasen, Shanghai, China) and 1% penicillin–streptomycin (PS, 15140122, Gibco), and cultured at 37 °C in a humidified incubator with 5% CO2 (Thermo Fisher Scientific, Waltham, MA, USA).

### 4.7. Plasmid Construction and Transient Transfection

MSCV-Puro-mCherry was applied as the plasmid vector to construct the *Tspo* plasmids. *Tspo* plasmids were decorated with V5 (GKPIPNPLLGLDST) tag in the C-terminus, and synthesized by Synbio Technologies Co., Ltd. (Suzhou, China). The codon and amino acid sequences were listed in Table S4. MSCV2.2-GFP vector was used to express *TLR4*, *TLR7* and *TLR9* plasmids, and HA (YPYDVPDYA) tags were fused to the C-terminal end of TLRs plasmids [42]. Transient transfection in HEK293T cells was performed using EZ Cell Transfection Reagent (AC04L092, Life-iLab, Shanghai, China).

### 4.8. Generation of Tspo^−/−^ BV2 and RAW 264.7 Cell Lines Using CRISPR-Cas9 Technology

The EGFP-tagged CRISPR plasmid pX330-U6-Chimeric_BB-CBh-hSpCas9 was kindly provided by Professor Liu Bo’s laboratory. Single-guide RNA (sgRNA)-targeting *Tspo* genes were designed using the CHOPCHOP online tool (http://chopchop.cbu.uib.no), and synthesized by Sangon Biotech (Shanghai, China, Table S5). Recombinant CRISPR-Cas9-*Tspo*-sgRNA plasmids were transformed into DH5α competent Escherichia coli (DLC101-100, MasterBio, Wuhan, China) and cultured overnight at 37 °C. Plasmids were subsequently extracted and purified using the TIANpure Midi Plasmid Kit (DP106, TIANGEN, Beijing, China). For generation of *Tspo* knockout (KO, *Tspo*^−/−^) cells, the CRISPR-Cas9-*Tspo*-sgRNA plasmids were transfected into WT cells using Lipofectamine™ 3000 (L3000001, Invitrogen, Waltham, MA, USA). Forty-eight hours post-transfection, EGFP-positive single cells were isolated by fluorescence-activated cell sorting (FACS; BD AriaFusion III, San Jose, CA, USA) and seeded into sterile 96-well plates. Monoclonal cell populations were expanded and the successful KO of TSPO was confirmed by Western blot.

### 4.9. Generation of RAW 264.7 Cell Lines with TSPO-V5 Overexpression

To generate TSPO-rescued RAW 264.7 cell lines, WT RAW 264.7 cells were transduced with lentiviral vectors expressing TSPO-V5. Briefly, HEK293T cells were co-transfected with MSCV-Puro-mCherry vectors carrying TSPO-V5 and lentiviral packaging plasmids. At 48 h after transfection, viral supernatants were collected, filtered through 0.45 μm syringe filters (SLHVR33RB, Merck Millipore, Billerica, MA, USA), and used to infect *Tspo*^+/+^ RAW 264.7 cells in the presence of polybrene (1:1000; C0351, Beyotime, Shanghai, China). Following infection, cells were maintained under standard culture conditions (37 °C, 5% CO_2_) and allowed to recover and proliferate for 48 h. Subsequently, transduced cells were selected with puromycin (4 μg/mL, HY-B1743, MCE, Monmouth Junction, NJ, USA) for 7−10 days to establish stable TSPO-overexpressing cell lines. The resulting rescued cell line was designated as *Tspo*^+/+^ + TSPO-V5.

### 4.10. Cell Stimulation

WT and *Tspo*^−/−^ BV2 cells were primed with 50 ng/mL LPS (L2880; Sigma-Aldrich, St. Louis, MO, USA) for 4 h and harvested for intracellular cytokine staining (ICS) to detect cytokine levels. *Tspo*^+/+^, *Tspo*^−/−^, and *Tspo*^+/+^ + TSPO-V5 RAW 264.7 cells were stimulated with LPS (100 ng/mL, 1 h), R848 (15 ng/mL, 6 h; tlrl-r848-10, InvivoGen, San Diego, CA, USA), or CpG-B (200 nM, 6 h; tlrl-1668-1, InvivoGen, San Diego, CA, USA). Culture supernatants were collected for quantification of TNF-α. For calcium intervention experiments, 10 mM anhydrous calcium chloride (CaCl_2_, HY-Y0406E, MCE, Monmouth Junction, NJ, USA) or 20 μM BAPTA-AM (HY-100545, MCE, Monmouth Junction, NJ, USA) was applied to WT and *Tspo*^−/−^ BV2 cells. For all experiments, non-stimulated (NS) control cells received an equal volume of dimethyl sulfoxide (DMSO, D8418, Sigma, St. Louis, MO, USA) or PBS (C0221A, Beyotime, Shanghai, China) as vehicle controls.

### 4.11. Enzyme-Linked Immunosorbent Assay (ELISA)

Concentrations of TNF-α in cell culture supernatants were quantified using a Mouse TNF-α ELISA kit (EM0001, HUABIO, Hangzhou, China), following the manufacturer’s instructions. Absorbance was read at 450 nm on a Synergy H1 microplate reader (BioTek, Winooski, VT, USA), and cytokine concentrations were calculated based on standard curves.

### 4.12. Co-Immunoprecipitation (Co-IP)

Co-IP assay was performed to assess the interaction of TSPO with TLRs proteins. Cells were lysed on ice in 1 mL of lysis buffer supplemented with EDTA-free complete protease inhibitor cocktail (HY-K0010, MCE) and 1mM PMSF (ST506, Beyotime). Lysates were incubated at 4 °C for 1 h with end-over-end rotation, followed by centrifugation (12,000 rpm, 30 min) to remove insoluble debris. A 100 μL aliquot of cell lysates was transferred to a fresh tube and mixed with 1× SDS-PAGE buffer to serve as input samples. The remaining lysates were incubated overnight at 4 °C with an anti-HA affinity matrix (11815016001, Roche, Basel, Switzerland), which had been pre-blocked with 1% BSA in lysis buffer for at least 30 min. The beads were subsequently washed five times with 1 mL lysis buffer to remove unbound proteins. Bead-bound proteins were eluted by incubation with 2× SDS-PAGE loading buffer at room temperature (RT) for 1 h with gentle agitation to serve as immunoprecipitated (IP) samples.

### 4.13. Western Blot

BV2 cell lysates were prepared following stimulation with 50 ng/mL LPS for the indicated time points (0, 15, 30, 60, 90, and 120 min). After treatment, cells were lysed on ice in 200 μL of 1× SDS-PAGE loading buffer per well supplemented with PhosSTOP™ EASYpack (04906837001, Roche), protease inhibitor cocktail, and 1mM PMSF. Lysates were collected into fresh tubes and boiled at 100 °C for 10 min to denature proteins. Equal amounts of protein were separated by SDS-PAGE and trans-blotted onto polyvinylidene difluoride (PVDF) membranes (IPVH00010, Merck Millipore). Membranes were blocked with 5% non-fat milk in Tris-buffered saline containing 0.1% Tween 20 (TBST) at RT for 1 h. After blocking, membranes were washed three times with TBST and incubated with primary antibodies overnight at 4 °C with gentle rotation. Following washing, PVDF membranes were incubated with appropriate secondary antibodies at RT for 1.5 h. Protein bands were visualized using enhanced chemiluminescence (ECL) reagents (SQ201, Epizyme, Cambridge, MA, USA) and detected using a LAS-3000 imaging system (Fujifilm, Tokyo, Japan). Band intensities were quantified by densitometric analysis using ImageJ software (version 1.54). Details of antibodies used for Western blot were provided in Table S6.

### 4.14. Intracellular Cytokine Staining (ICS)

For ICS assay, cells were treated with LPS for 30 min, followed by the addition of brefeldin A (10 μg/mL; 555029, BD Biosciences, San Jose, CA, USA) to inhibit cytokine secretion, and incubated for an additional 4 h. Cells were then harvested, resuspended as single-cell suspensions, and stained with LIVE/DEAD Fixable Violet Dead Cell Stain (L34964, Invitrogen) in 96-well U-bottom plates at 4 °C for 20 min to exclude non-viable cells. After washing twice with FACS buffer (PBS containing 2% FBS and 1% PS), cells were fixed and permeabilized using the Cytofix/Cytoperm™ Kit (554714, BD Biosciences). Fc receptors were subsequently blocked by incubation with anti-CD16/32 antibody (553142, BD Biosciences) for 30 min at 4 °C in dark. Fluorochrome-conjugated antibodies were diluted in Perm/Wash buffer and incubated with cell pellets for 30 min at 4 °C in dark. Following staining, cells were washed and resuspended in ice-cold FACS buffer, and analysis was performed on a BD Fortessa flow cytometer. The mean fluorescence intensity (MFI) was calculated based on the geometric mean using FlowJo software (v10.9.0). Antibodies used for ICS were listed in Table S7.

### 4.15. Measurement of Intracellular Ca^2+^ Levels

Cytosolic Ca^2+^ levels were measured by the Fluo-4 Calcium Assay Kit (S1061S, Beyotime), and mitochondrial Ca^2+^ were detected by the Calcium Assay Kit with Rhod-2 AM (S1062S, Beyotime). Calcium Assay Kits were conducted in accordance with the manufactural protocols, and the Ca^2+^ intensity was detected by BD Fortessa and MFI was analyzed based on the geometric mean.

### 4.16. Cholesterol Uptake Assay Using BODIPY–Cholesterol

The cellular cholesterol uptake was evaluated utilizing the BODIPY–cholesterol-based Cholesterol Uptake Assay Kit (S0603M, Beyotime) in accordance with the manufacturer’s protocol. Fluorescent micrographs were acquired on an inverted fluorescence microscope (Olympus CKX5, Tokyo, Japan). Quantitative fluorescence measurements were performed on a microplate reader with excitation at 485 nm and emission at 528 nm. Intergroup analysis of relative fluorescence units (RFU) was implemented to quantify the cellular cholesterol uptake capacity. All manipulations were conducted under dark conditions to mitigate photobleaching.

### 4.17. Measurement of Intracellular ATP and L-Lactate Contents

An Enhanced ATP Assay Kit (S0027, Beyotime) was used to detect the cellular ATP concentrations. Intracellular L-lactate, one of the end-products of both anaerobic and aerobic glycolysis [43,44], was determined by using the L-Lactate Assay Kit with WST-8 (S0208S, Beyotime). Both ATP and L-lactate assays were finished according to the manufactural protocol. Total cellular ATP and L-lactate contents were calculated based on the standard curve and normalized by the baseline concentrations.

### 4.18. Molecular Docking Analysis

Molecular docking was used to predict the potential conformation regarding the murine TSPO and TLR4 interaction. Three-dimensional structures of TSPO (P50637) and TLR4 (Q9QUK6) proteins were obtained from the UniProt database. The binding interfaces of the TLR4-TSPO complexes were systematically analyzed, and the docking model with the highest affinity was adopted. The interaction interfaces were further analyzed using LigPlot+ (version 2.3), which provided the intermolecular interactions involved with amino acid residues, hydrogen bonds and hydrophobic contacts. PyMOL software (version 4.3.0) was used to analyze the three-dimensional interaction details and visualize the docking model. Amino acid residues involved in interactions within a distance of ≤5Å were identified for further analysis.

### 4.19. Statistical Analysis

Statistical analyses were conducted using R software (version 4.6.1), GraphPad prism (version 9.5) and SPSS Statistics (version 27.0). Quantitative data were presented as mean ± standard deviation (M ± SD). Comparisons between two independent groups were conducted using an unpaired two-tailed Student’s *t*-test. For experiments involving two independent variables, two-way analysis of variance (ANOVA) followed by appropriate post hoc tests was performed. The assumptions of normal distribution and homogeneity of variance were evaluated before applying parametric statistical tests. All in vitro experiments were performed with at least three independent biological replicates. ROC curve analysis was performed to assess the diagnostic performance of *TSPO* and inflammatory genes, including *IL1B*, *IL6*, *IL17A*, *IL18*, and *TNF*. The area under the ROC curve (AUC) with 95% confidence intervals (CIs) was calculated, and optimal cut-off values were determined using Youden’s index. Differences between ROC curves were compared using DeLong’s test. A two-sided *p* value < 0.05 was considered statistically significant. Statistical significance was denoted as * *p* < 0.05, ** *p* < 0.01, *** *p* < 0.001, and **** *p* < 0.0001; ns indicated not significant.

## 5. Conclusions

In summary, this study identifies TSPO as an important immunometabolic regulator that integrates TLR4-mediated inflammatory signaling with Ca^2+^ regulation, cholesterol homeostasis, and metabolic adaptation during bacterial infection. Rather than serving merely as a marker of inflammatory activation, TSPO functions as a regulatory component that selectively constrains excessive TLR4-driven inflammatory responses while maintaining cellular metabolic homeostasis. Beyond its mechanistic role, TSPO demonstrates strong diagnostic potential for distinguishing bacterial infection, highlighting its potential clinical utility.

## Data Availability

Transcriptomic data are publicly available at GEO (geo@ncbi.nlm.nih.gov) under accession number (GSE72829). Experimental datasets presented in the current study are available from the corresponding author upon reasonable request.

## Supplementary Materials

The following supporting information can be downloaded at: https://www.mdpi.com/article/10.3390/ijms27167336/s1.

## Abbreviations

AUC	area under the curve
BP	biological process
CaCl_2_	calcium chloride anhydrous
CC	cellular component
CNS	central nervous system
CI	confidence interval
Co-IP	co-immunoprecipitation
DEG	differentially expressed gene
DMEM	Dulbecco’s modified Eagle medium
DMSO	dimethyl sulfoxide
FACS	fluorescence activated cell sorting
FBS	fetal bovine serum
GEO	Gene Expression Omnibus
GO	Gene Ontology
GSEA	Gene Set Enrichment Analysis
HC	healthy control
HIF-1	hypoxia-inducible factor 1
ICS	intracellular cytokine staining
IL-6	interleukin-6
IP	immunoprecipitated
KEGG	Kyoto Encyclopedia of Genes and Genomes
KO	knockout
log_2_FC	log_2_ fold change
LPS	lipopolysaccharide
MAPK	mitogen-activated protein kinase
MF	molecular function
MFI	mean fluorescence intensity
MSigDB	Molecular Signatures Database
|NES|	absolute normalized enrichment score
NF-κB	nuclear factor kappa B
PBS	phosphate-buffered saline
PMSF	phenylmethylsulphonyl fluoride
PS	penicillin-streptomycin
PVDF	immobilon polyvinylidene difluoride
ROC	receiver operating characteristic
ROS	reactive oxygen species
RT	room temperature
sgRNA	single-guide RNA
ssGSEA	single-sample gene set enrichment analysis
TBST	Tris-buffered saline with 0.1% Tween 20
TLR	Toll-like receptor
TNF-α	tumor necrosis factor-α
TSPO	Translocator protein 18 kDa
