# Supplementary material for: TSPO Regulates TLR4-Mediated Inflammation Through Calcium Homeostasis and Immunometabolic Adaptation
Source: Int J Mol Sci. 2026 Aug 17;27(16):7336. doi: 10.3390/ijms27167336 (PMC13513882; doi:10.3390/ijms27167336)
Supplement: Supplementary file 1 [file ijms-27-07336-s001.zip › Legends for supplementary figures.pdf]

Figure S1. Transcriptomic comparison between bacterial and viral infection cohorts.

(**A**) Volcano plot illustrating differentially expressed genes (DEGs) in bacterial infection versus viral infection groups, screened with thresholds of $p<1\times10^{-5}$ and $|\log_2FC| > 0.5$. Upregulated (red), downregulated (blue), and non-significant (gray) genes were indicated. (**B−D**) Violin plots comparing *TLR7*, *TLR8* and *TLR9* mRNA expression across bacterial infection, viral infection and healthy control groups. Transcriptomic data were obtained from the GSE72829 dataset. ** $p<0.01$; **** $p<0.0001$; ns, not significant.

Figure S2. Comparisons of ssGSEA scores of hallmark pathway between definite bacterial infection (*n*=52) and healthy contro (HC) group (*n*=16).

(**A**) Violin plots comparing ssGSEA enrichment scores of inflammation-related hallmark pathways, including inflammatory response, IL-6/JAK/STAT3 signaling, and TNF-α signaling via NF-κB, in bacterial infection patients versus HCs. (**B**) Violin plots comparing ssGSEA enrichment scores of metabolism- and stress-related hallmark pathways, including reactive oxygen species (ROS) pathway, cholesterol homeostasis, xenobiotic metabolism, and p53 pathway, between bacterial infection patients and HCs. (**C**) Scatter plots depicting positive correlations between TSPO expression and ssGSEA scores of inflammatory response, IL-6/JAK/STAT3 signaling, and TNF-α signaling via NF-κB. Data were retrieved from the GSE72829 dataset. ***$p < 0.001$, ****$p < 0.0001$; ns, not significant.

Figure S3. Structural basis and selective interaction of TSPO with TLR4.

(**A**) LigPlot+ analysis illustrating the intermolecular interaction interface between murine TLR4 and murine TSPO. The plot depicting key amino acid residues (chain identifiers) involved in binding interface stabilized by hydrogen bonds (green dashed lines) and hydrophobic interactions (red arc and radiating lines). (**B, C**) Co-IP assay confirming the absence of interaction between murine TSPO-V5 with (B) TLR7-HA and (C) TLR9-HA.

Figure S4. Cell-type-specific expression patterns of *TLR4* and *TSPO* in human and mouse brain and peripheral blood.

(**A, B**) Expression levels of (**A**) *TLR4* and (**B**) *TSPO* across distinct cell populations in healthy human brain. (**C, D**) Cell-type expression profiles of (**C**) *Tlr4* and (**D**) *Tspo* in mouse brain. (**E,**

F) Cellular distribution of *TSPO* transcripts in (**E**) human and (**F**) mouse peripheral blood. Cell-type-resolved transcriptomic data were obtained via the CZ CELLxGENE Discover platform.

Figure S5. Cross-species conserved co-expression of *TSPO* and TLR4-associated anti-inflammatory genes in brain cells.

(A−D) Scatter plots illustrating Spearman correlations between *TSPO* and (A) *TLR4*, (B) *HMOX1*, (C) *ARG1*, (D) *IL10* in human brain cells. (E−H) Spearman correlation analyses of *Tspo* with (E) *Tlr4*, (F) *Hmox1*, (G) *Arg1*, and (H) *Il10* in mouse brain single-cell datasets. Transcriptomic data were obtained from the CZ CELLxGENE Discover platform. $^{**}p < 0.01$, $^{***}p < 0.001$, $^{****}p < 0.0001$.

Figure S6. Identification of the TSPO knockout and TSPO overexpression in cell lines.

Western blot detecting the knockout of TSPO in (**A**) BV2 and (**B**) RAW 264.7 cell lines. (**C**) The overexpression of TSPO-V5 and knockout of TSPO in RAW 264.7 macrophages, as compared with WT RAW 264.7 cells.

Figure S7. Representative BODIPY-cholesterol fluorescence staining for intracellular cholesterol detection.

(**A**) Fluorescence images showing cholesterol labeling in *Tspo*$^{+/+}$ and *Tspo*$^{-/-}$ BV2 microglia. (**B**) BODIPY-cholesterol staining in *Tspo*$^{+/+}$, *Tspo*$^{-/-}$, and *Tspo*$^{+/+}$+TSPO-V5 overexpressing RAW 264.7 macrophages. Green: BODIPY-cholesterol; blue: Hoechst 33342 nuclear stain. All images were acquired with an Olympus CKX5 inverted fluorescence microscope. Scar bars, 20 μm.

Figure S8. KEGG and GO enrichment of *TSPO*-correlated genes derived from transcriptome datasets of patients with bacterial infection.

(A) Bubble plot of KEGG analysis identified enriched pathways including the Toll-like receptor signaling pathway, HIF-1 signaling pathway, phagosome, endocytosis and lysosome biogenesis, indicative of coordinated innate immune and immunometabolic activation. (B) GO enrichment bubble plot separated into Biological Process (BP), Cellular Component (CC) and Molecular Function (MF) categories. BP terms reflected innate immune activation and leukocyte inflammatory responses; CC terms were enriched for phagolysosomal compartments and secretory vesicles; MF terms featured Toll-like receptor binding and immune receptor activity,

indicative of enhanced inflammatory signal transduction and energy requirements. Transcriptomic data were obtained from the GSE72829 dataset.
