# Supplementary material for: TSPO Regulates TLR4-Mediated Inflammation Through Calcium Homeostasis and Immunometabolic Adaptation
Source: Int J Mol Sci. 2026 Aug 17;27(16):7336. doi: 10.3390/ijms27167336 (PMC13513882; doi:10.3390/ijms27167336)
Supplement: Supplementary file 1 [file ijms-27-07336-s001.zip › Supplementary Table S1.pdf]

Table S1. Spearman correlation of TSPO expression with key molecules involved in inflammatory and regulatory signaling pathways in HC group (*n*=16).

	TSPO			***TSPO***	
**Items**	***r***	***p* value**	**Items**	***r***	***p* value**
TLR4	0.311	0.2406	***HMOX1***	0.338	0.2008
***NFKBIA*(IκBα)**	0.523	0.0375*	***ARG1***	0.053	0.8447
TNF	0.195	0.4699	***IL10***	−0.264	0.3223
IL6	−0.214	0.4255	***ATP6AP1***	0.378	0.1486
CAMKK2	0.096	0.7249	***ATP6V0A1***	0.209	0.4369
CAMK2D	−0.246	0.3580	***ATP6V0D2***	−0.286	0.2834
CAMK2G	0.476	0.0624	***ATP6V1A***	0.043	0.8753
