# Supplementary material for: TSPO Regulates TLR4-Mediated Inflammation Through Calcium Homeostasis and Immunometabolic Adaptation
Source: Int J Mol Sci. 2026 Aug 17;27(16):7336. doi: 10.3390/ijms27167336 (PMC13513882; doi:10.3390/ijms27167336)
Supplement: Supplementary file 1 [file ijms-27-07336-s001.zip › Supplementary Table S2.pdf]

Table S2. Spearman correlation of TSPO expression with key molecules involved in inflammatory and regulatory signaling pathways in patients with definite bacterial infection (*n*=52).

	TSPO			***TSPO***	
**Items**	***r***	***p* value**	**Items**	***r***	***p* value**
TLR4	0.682	<0.0001	***NFKBIA*(IκBα)**	0.675	<0.0001
TLR7	−0.448	<0.0001	***ARG1***	0.621	<0.0001
TLR9	0.095	0.5011	***IL10***	0.846	<0.0001
***NFKB1* (p50)**	0.459	0.0006	***HMOX1***	0.671	<0.0001
***MAPK9* (JNK2)**	−0.355	0.0097	***ATP6AP1***	0.790	<0.0001
***MAPK14* (p38α)**	0.324	0.0189	***ATP6V0A1***	0.719	<0.0001
***MAPK12* (p38γ)**	−0.274	0.0497	***ATP6V0D1***	0.802	<0.0001
TNF	0.536	<0.0001	***ATP6V1A***	0.535	<0.0001
IL6	0.658	<0.0001	***CAMK2D***	−0.386	<0.0001
IL1B	0.548	<0.0001	***CAMKK2***	0.663	<0.0001
