# Supplementary material for: TSPO Regulates TLR4-Mediated Inflammation Through Calcium Homeostasis and Immunometabolic Adaptation
Source: Int J Mol Sci. 2026 Aug 17;27(16):7336. doi: 10.3390/ijms27167336 (PMC13513882; doi:10.3390/ijms27167336)
Supplement: Supplementary file 1 [file ijms-27-07336-s001.zip › Supplementary Table S3.pdf]

Table S3. Diagnostic performance of *TSPO* and inflammatory genes in the identification of bacterial infection.

		ROC curve analysis					DeLong's test		
		**AUC**	***p* value**	**Cut Point**	**Sensitivity, %**	**Specificity, %**	**Z score**	***p* value**	**AUC difference (95% CI)**
**Differentiation of bacterial from viral infection**	*TSPO*	0.849 (0.774−0.924)	*p*<0.0001	>12.090	73.08	90.22	NIS	NIS	NIS
	IL1B	0.531 (0.434−0.629)	*p*=0.5327	<9.759	51.92	60.87	7.751	*p*<0.0001	0.380 (0.284−0.477)
	IL6	0.662 (0.573−0.751)	*p*=0.0013	>9.175	78.85	48.91	4.152	*p*<0.0001	0.187 (0.099−0.275)
	IL17A	0.578 (0.480−0.676)	*p*=0.1198	>5.279	71.15	46.74	4.304	*p*<0.0001	0.271 (0.148−0.394)
	IL18	0.582 (0.485−0.679)	*p*=0.1030	<14.31	75.00	46.74	7.186	*p*<0.0001	0.431 (0.313−0.549)
	TNF	0.6737 (0.573−0.774)	*p*=0.0005	>8.601	51.92	83.70	3.499	*p*=0.0005	0.175 (0.077−0.274)
**Differentiation of bacterial infection from HCs**	*TSPO*	0.999 (0.995−1.000)	*p*<0.0001	>9.890	98.08	100.00	NIS	NIS	NIS
	IL1B	0.870 (0.787−0.954)	*p*<0.0001	>9.454	67.31	100.00	3.045	*p*=0.0023	0.129 (0.046−0.211)
	IL6	1.000 (1.000−1.000)	*p*<0.0001	>8.358	100.00	100.00	−0.707	*p*=0.4795	−0.001 (−0.005−0.002)
	IL17A	0.856 (0.767−0.945)	*p*<0.0001	<5.546	73.08	100.00	18.690	*p*<0.0001	0.855 (0.765−0.944)
	IL18	1.000 (1.000−1.000)	*p*<0.0001	>13.09	100.00	100.00	−0.707	*p*=0.4795	−0.001 (−0.005−0.002)
	TNF	0.974 (0.943−1.000)	*p*<0.0001	>7.297	90.38	100.00	1.697	*p*=0.0897	0.025 (−0.004−0.054)

Note: NIS, not in statistics.
