# Supplementary material for: TSPO Regulates TLR4-Mediated Inflammation Through Calcium Homeostasis and Immunometabolic Adaptation
Source: Int J Mol Sci. 2026 Aug 17;27(16):7336. doi: 10.3390/ijms27167336 (PMC13513882; doi:10.3390/ijms27167336)
Supplement: Supplementary file 1 [file ijms-27-07336-s001.zip › Supplementary Table S4.pdf]

Table S4. Amino acid sequences of TSPO-V5.

Item	Amino acid sequence
TSPO-V5	AGATCTGCCACCATGCCTGAATCCTGGGTGCCTGCCGTGGGCCTCACT<br>CTGGTGCCCAGCCTGGGGGGCTTCATGGGAGCCTACTTTGTACGTGGC<br>GAGGGCCTCCGGTGGTATGCTAGCTTGCAGAAACCCTCTTGGCATCCG<br>CCTCGCTGGACACTGGCTCCCATCTGGGGCACACTGTATTCAGCCATG<br>GGGTATGGCTCCTACATAGTCTGGAAAGAGCTGGGAGGTTTCACAGAG<br>GACGCTATGGTTCCCTTGGGTCTCTACACTGGTCAGCTGGCTCTGAACT<br>GGGCGTGGCCCCCCATCTTCTTTGGTGCCCGGCAGATGGGCTGGGCCT<br>TGGCCGATCTTCTGCTTGTCAGTGGGGTGGCGACTGCCACAACCCTGG<br>CTTGGCACCGAGTGAGCCCGCCGGCTGCCCGCTTGCTGTACCCTTACC<br>TGGCCTGGCTGGCTTTTGCCACCGTGCTCAACTACTATGTATGGCGTGA<br>TAACTCTGGCCGGCGAGGGGGCTCCCGGCTCCCAGAGGGTAAGCCTAT<br>CCCTAACCCTCTCCTCGGTCTCGATTCTACGTGACTCGAG
