# Supplementary material for: TSPO Regulates TLR4-Mediated Inflammation Through Calcium Homeostasis and Immunometabolic Adaptation
Source: Int J Mol Sci. 2026 Aug 17;27(16):7336. doi: 10.3390/ijms27167336 (PMC13513882; doi:10.3390/ijms27167336)
Supplement: Supplementary file 1 [file ijms-27-07336-s001.zip › Supplementary Table S5.pdf]

Table S5. Primers used for the construction of *Tspo*-sgRNA.

**Items**		**Primer sequence (5' to 3')**
***Tspo*-sgRNA1**	F	5'-TACGTGGCGAGGGCCTCCGG-3'
	R	5'-CCGGAGGCCCTCGCCACGTA-3'
***Tspo*-sgRNA2**	F	5'-GCCCTCGCCACGTACAAAGT-3'
	R	5'-ACTTTGTACGTGGCGAGGGC-3'
