# Supplementary material for: TSPO Regulates TLR4-Mediated Inflammation Through Calcium Homeostasis and Immunometabolic Adaptation
Source: Int J Mol Sci. 2026 Aug 17;27(16):7336. doi: 10.3390/ijms27167336 (PMC13513882; doi:10.3390/ijms27167336)
Supplement: Supplementary file 1 [file ijms-27-07336-s001.zip › Supplementary Table S6.pdf]

Table S6. Antibodies used in Western blot assay.

Antibodies	Source	Identifier
**Primary antibodies**		
TSPO	Abmart	T55408S
V5 tag	Abmart	TT0057S
Anti-HA High Affinity (3F10)	Roche	11867423001
p38 MAPK	CST	9212
Phospho-p38 MAPK (Thr180/Tyr182)	CST	9211
JNK2 (56G8)	CST	9258
Phospho-SAPK/JNK (Thr183/Tyr185) (81E11)	CST	4668
IκBα	CST	9242
Tubulin	Abmart	M20005
GAPDH	Proteintech	60004-1-Ig
**Secondary antibodies**		
Donkey Anti-Rabbit IgG (H+L)	Jackson	711-165-152
Goat Anti-Mouse IgG (H+L)	Jackson	115-035-003
Donkey Anti-Rat IgG (H+L)	Jackson	712-165-150
