# Supplementary material for: TSPO Regulates TLR4-Mediated Inflammation Through Calcium Homeostasis and Immunometabolic Adaptation
Source: Int J Mol Sci. 2026 Aug 17;27(16):7336. doi: 10.3390/ijms27167336 (PMC13513882; doi:10.3390/ijms27167336)
Supplement: Supplementary file 1 [file ijms-27-07336-s001.zip › Supplementary Table S7.pdf]

Table S7. Antibodies used in ICS assay.

Antibodies	Source	Identifier
APC anti-mouse TNF-α Antibody	Biolegend	506307
PE anti-mouse IL-6 Antibody	Biolegend	504503
